# Synthesis and Surface Engineering of Two‐Dimensional MXenes for Advanced Functional Applications

**DOI:** 10.1002/tcr.70194

**Published:** 2026-06-25

**Authors:** Dalil N. H. Al‐Ghubairi, Hadi M. Marwani, Mahmood D. Aljabri, Muhammad Fazle Rabbee, Mohammed Muzibur Rahman

**Affiliations:** ^1^ Chemistry Department, Faculty of Science King Abdulaziz University Jeddah Saudi Arabia; ^2^ Center of Excellence for Advanced Materials Research King Abdulaziz University Jeddah Saudi Arabia; ^3^ Department of Chemistry University College in Al‐Jamoum, Umm Al‐Qura University Makkah Saudi Arabia; ^4^ Department of Biotechnology Yeungnam University Gyeongsan Republic of Korea

**Keywords:** 2D MXenes, catalysis, CVD, energy storages, MAX phases, sensors

## Abstract

Two‐dimensional MXenes (2DMxn), a rapidly expanding family of transition metal carbides, nitrides, and carbonitrides with the general formula (M_*n* + 1_X_*n*_T_*x*_), have attracted significant attention for their metallic conductivity, hydrophilic surfaces, high surface area, and tunable surface terminations. These unique properties make MXenes promising candidates for applications in catalysis, sensing, energy storage, and environmental remediation. This review provides a comprehensive overview of recent advances in MXene research, particularly developments reported since 2020. The discussion begins with synthesis strategies, including conventional top‐down etching of MAX phases using hydrofluoric acid and in situ fluoride‐based methods, as well as safer fluoride‐free approaches such as electrochemical and hydrothermal techniques and Lewis‐acid molten‐salt etching. Emerging bottom‐up fabrication routes, including chemical vapor deposition (CVD) and template‐assisted growth, are also highlighted for their potential to provide improved structural control. Furthermore, advanced postsynthesis modification methods such as solvothermal and microwave‐assisted treatments, atomic layer deposition (ALD), electrochemical engineering, and ball‐milling are discussed as effective strategies for tailoring interlayer spacing, surface chemistry, and hierarchical architectures. The review also summarizes the recent progress of MXene‐based materials in photocatalysis, electrocatalysis, energy storage devices, and electrochemical sensors, with particular emphasis on structure–property–performance relationships that govern charge transfer, active‐site accessibility, and stability. Finally, key challenges including oxidation susceptibility, nanosheet restacking, and the need for scalable and safe production methods are critically discussed, together with future prospects for the development of MXene‐based multifunctional materials and devices for next‐generation technologies.

## Graphical abstract

This study highlights the synthesis, surface engineering, and advanced functional applications of MXenes, emphasizing key preparation strategies that enable their use in catalysis, energy storage, sensing, and environmental remediation.

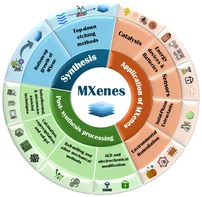

## 1 Introduction

**FIGURE 1:**
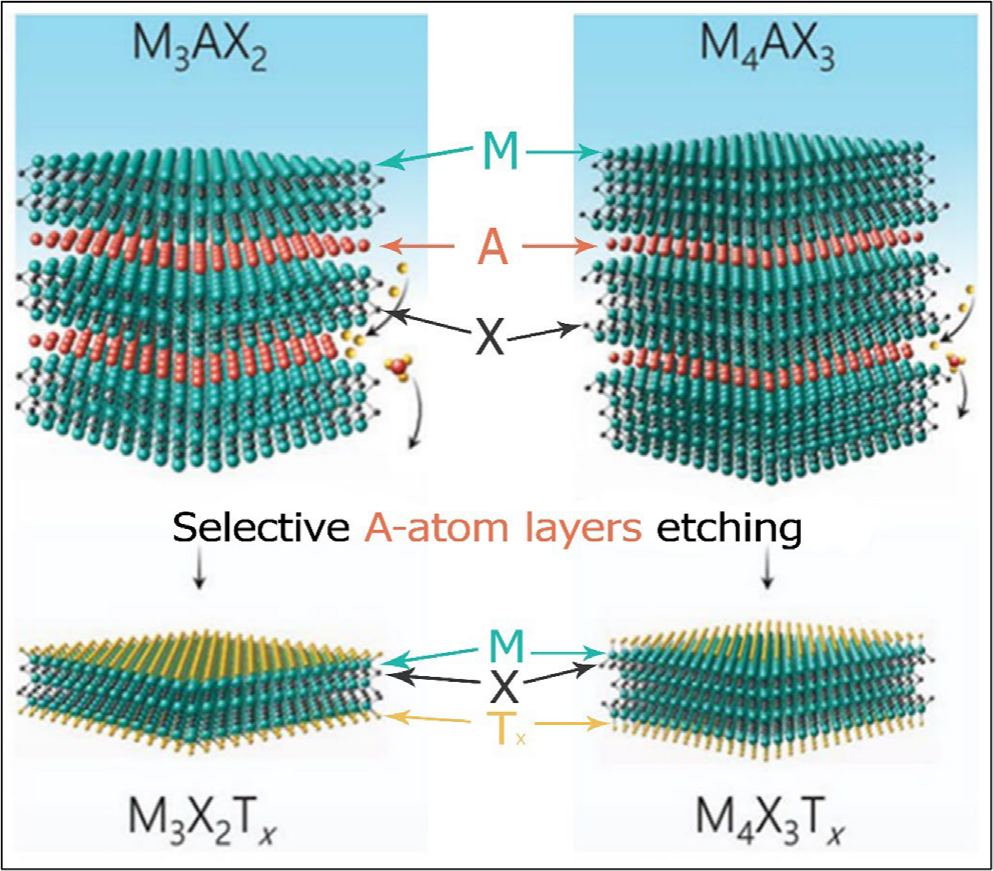
Diagram shows how to make different MXenes from MAX phases. Adapted from [2] published under Creative Commons Attribution 4.0 International License (CC BY 4.0).

Nanomaterials have emerged as a cornerstone of modern materials research, owing to their distinct physicochemical features and extensive application possibilities, particularly in sensing, energy, and biological domains. Two‐dimensional (2D) materials, such as MXenes, have attracted significant interest due to their huge surface area, adjustable electrical structures, and high mechanical strength, making them highly promising for sophisticated sensing technologies with high sensitivity, rapid response, and improved selectivity [1]. Furthermore, their metallic‐like electrical conductivity, adjustable interlayer spacing, low ion diffusion barrier, and rich surface chemistry make them suitable for energy storage and conversion, optoelectronic devices, electromagnetic interference shielding, environmental applications, biomedical applications [2, 3], and biosensing and point‐of‐care diagnostics [4]. MXenes are made up of transition metal carbides, nitrides, or carbonitrides with the general formula (M_*n* + 1_ X*_n_* T*_x_*). M symbolizes a transition metal (e.g., Ti, V, Mo, or W), X is carbon or nitrogen, and T*_x_* signifies surface termination groups such as —O, —OH, and —F [1], which are directly controlled by the synthesis route and have a significant impact on the electronic structure and properties of MXenes [5]. Over the last 15 years, more than 40 MXene compositions have been synthesized since Gogotsi et al.'s 2011 discovery of Ti_3_C_2_T*_x_* [1]. MXenes are formed by the selective extraction of A‐layer atoms (like Al or Si) from their layered MAX phase precursors. In this process, the weaker M—A bonds are chemically etched away, while the stronger M—X bonds stay intact. This results in loosely stacked 2D layers, as shown in Figure 1. Considering that M—A bonds are weaker than M—X bonds, the etchant species specifically target the A layer in the MAX phase during etching. Fluoride ions have a great affinity for Al in HF‐based pathways, which encourages the creation of AlF_3_ and the gradual loss of the A layer. Following the removal of the A layer, interactions with the etching media and water molecules cause the exposed transition‐metal surface to be terminated by —F, —OH, and —O groups. Layered MXene sheets are eventually formed as a result of this process, which also weakens interlayer contacts and increases interlayer separation [6].

MXenes have strong metallic conductivity [7], a huge specific surface area (>1000 m^2^/g) [8], hydrophilicity from surface terminations (—O, —OH, —F), and exceptional mechanical strength with elastic constants greater than MAX phases [9]. The nature and distribution of these terminations are strongly governed by the etching process since the choice of precursor, etchant, and reaction conditions directly determines the surface chemistry of the resulting MXene sheets. In particular, fluoride‐based etching routes often favor the formation of —F terminations, whereas alternative etching strategies can increase the relative abundance of —O and —OH groups, thereby altering the electronic structure and conductivity of MXenes [10]. Moreover, etching time is another critical parameter, as prolonged exposure may promote overetching, structural defects, and oxidative by‐products, while moderate conditions can preserve layered integrity and improve material quality [11]. Therefore, understanding how synthesis conditions control surface terminations provides the basis for discussing the different MXene preparation routes in the following section. Furthermore, the extraordinary features of MXenes stem from their unusual electronic structure and strong metal–carbon/nitrogen bonding, which allow for efficient electron transport and high charge carrier mobility. Their electronic properties can be carefully controlled via surface terminations, stoichiometric variation, and defect engineering, giving them control over conductivity and band structure. Mechanically, MXenes have high rigidity and significant Young's modulus values, owing to strong M—X bonding, while remaining flexible enough for composite integration. They have moderately high thermal conductivity, which is highly sensitive on composition, interlayer spacing, and surface chemistry. Furthermore, new research indicates that certain MXenes may exhibit composition‐ and termination‐dependent magnetic behavior, emphasizing their multifunctionality [12].

In addition to their scientific significance, MXenes are attracting increasing industrial interest due to their tunable properties, compatibility with scalable processing, and broad applicability across various technologies. However, despite this growing attention, several challenges remain, including difficulties in controlling surface terminations during etching, limited environmental stability (particularly under aqueous and oxidative conditions), and the reliance on hazardous chemicals such as hydrofluoric acid in conventional synthesis routes. Addressing these limitations is essential for advancing MXenes from laboratory research toward practical application [5].

Ti_3_C_2_T*_x_* MXene is a well‐studied MXene prototype due to its successful synthesis and easy etching and delamination methods [13]. Furthermore, its excellent electrical conductivity, customizable surface terminations, and high hydrophilicity make it a versatile platform for energy storage, sensing, and catalysis [14]. This dominance is clearly reflected in the literature reviewed in this review, where Ti‐based MXenes, particularly Ti_3_C_2_T_*x*_, account for approximately 70%–80% of the reported experimental studies. This study reviews recent advances in MXene synthesis and surface modification approaches, as well as promising applications in energy, catalysis, and sensing. A particular focus is placed on studies published after 2020 that examine how structural design and surface engineering influence functional performance. The review also discusses modern developments and future directions for MXene‐based materials.

## 2 Preparation of MXenes

### 2.1 MXene Synthesis Methods (Bottom‐Up versus Top‐Down)

**FIGURE 2:**
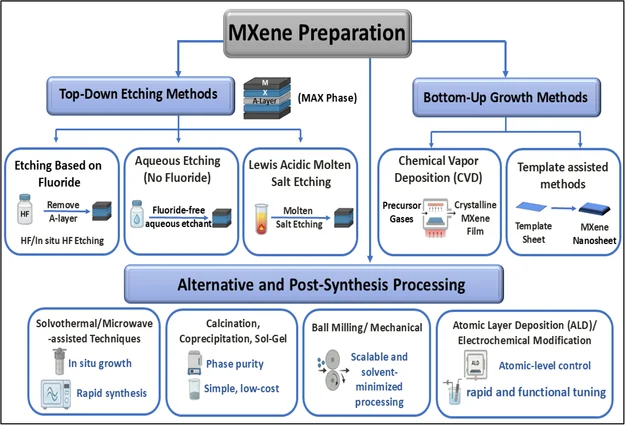
Flowchart outlining preparation techniques for MXene and postsynthesis processing pathways.

MXene synthesis routes have greatly influenced their electrical and physicochemical properties, as well as their applications [15]. In general, there are two primary approaches to MXene synthesis: top‐down methods that begin with layered MAX precursors and bottom‐up methods that directly create 2D transition‐metal carbides from basic inorganic feedstocks (Figure 2). In top‐down approaches, the parent MAX is transformed into multilayer MXene with a stacked lamellar architecture by first selectively etching bulk MAX‐phase ceramics to remove the A‐site element (such as Al or Si), usually using liquid‐phase etchants. This is followed by the intercalation of small molecules or ions and subsequent delamination, which is frequently aided by mechanical agitation or sonication, to produce few‐ or single‐layer MXene nanosheets with atomic‐scale thickness and nanoscale lateral dimensions. Because of their high surface area and accessible active sites, these nanosheets have been widely used in energy storage, sensing, and biomedical systems [16]. Bottom‐up synthesis, which grows MXene‐like 2D carbides directly instead of peeling them from a bulk parent phase, is a fundamentally distinct approach that complement existing exfoliation‐based techniques. These methods, such as chemical vapor deposition and wet‐chemical growth, create ultrathin transition‐metal carbide layers under carefully regulated temperature, environment, and growth time by combining metal substrates or metal/halide systems with carbon (or other) precursors. Large‐area ultrathin crystals with lower defect density and customized surface exposure can be created using these bottom‐up approaches, which offer atomic‐level control over composition, crystallinity, thickness, and lateral dimension. They are therefore especially appealing when direct liquid‐phase exfoliation is difficult, when nontraditional MXene compositions are sought after, or when unique structures are needed for high‐performance electrochemical, catalytic, and electrical devices [16, 17].

### 2.2 Top‐Down Etching Methods from MAX to MXene

Among these two approaches, top‐down liquid‐phase etching of MAX precursors remains the most widely used method for obtaining MXenes in large numbers [15]. However, a range of chemically unique etching media have since been created, which can be broadly classed as fluoride‐based, aqueous etching methods without fluoride, and Lewis acidic molten‐salt systems, each offering differing levels of safety, termination control, and structural stability.

#### 2.2.1 *Etching Based on Fluoride* (*HF and In Situ HF Systems*)

**FIGURE 3:**
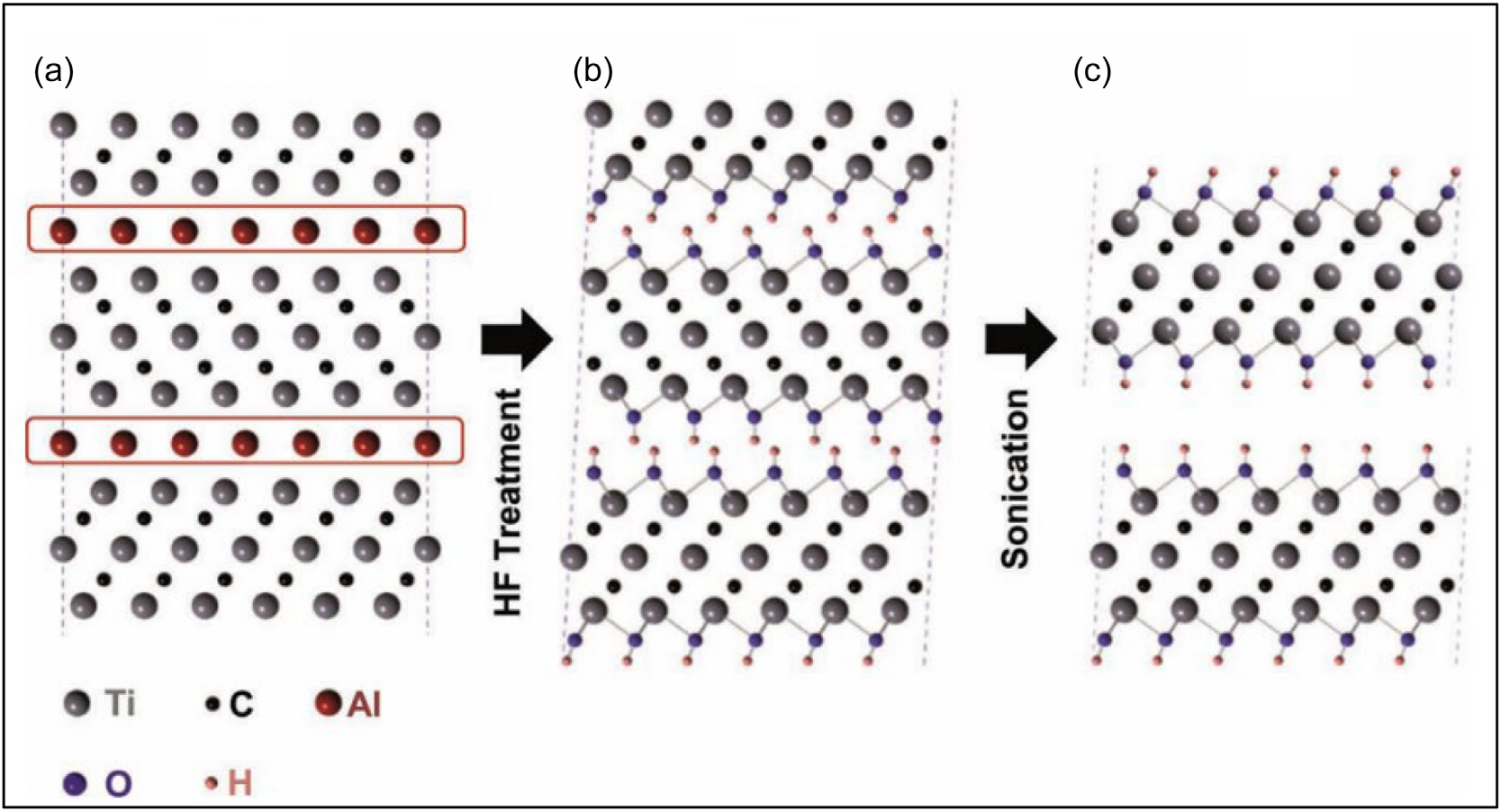
Ti_3_AlC_2_ (a) etching in HF (b) to yield Ti_3_C_2_T*_x_* MXene layers (c). Reproduced with permission [18] Copyright 2026, John Wiley & Sons (License No. 6264480901738).

Fluoride‐based etching has been the main technique for MXene production since the discovery of Ti_3_C_2_T*_x_* in 2011. This method uses hydrofluoric acid (HF) to selectively remove the A‐site element (typically Al) from the MAX phase (Ti_3_AlC_2_) while preserving the M_*n* + 1_X*_n_* framework as shown in Figure 3. The result is multilayer MXene with surface terminations including —OH and —F groups [18].

Ti_3_C_2_T*_x_* MXene is typically synthesized via a top‐down chemical etching route. A representative example is the microwave‐assisted HF etching of Ti_3_AlC_2_ MAX phase to produce Ti_3_C_2_T*_x_* MXene [19]. The Ti_3_AlC_2_ MAX phase is etched in concentrated HF to selectively remove the Al layers and produce layered Ti_3_C_2_T*_x_*, followed by postalkaline treatment in NaOH to adjust surface terminations and intercalate Na^+^ ions. While HF etching can provide organ‐like Ti_3_C_2_T*_x_* with a porous structure, further surface modification, such as alkaline treatment, is necessary to optimize surface terminations and fully use MXene for sensing applications. Alternative ways have been developed to create HF in situ from fluoride salts and mineral acids [20]. As reported in [21], a LiF/HCl system was used to selectively remove Al from the Ti_3_AlC_2_ MAX phase while forming MXene layers. This system produces HF constantly inside the reaction vessel. In situ etching is a safer and more controllable way to produce high‐quality Ti_3_C_2_T*_x_* nanosheets for large‐scale processing. In study [22], Ti_3_C_2_T*_x_* MXene nanosheets were first prepared by LiF/HCl etching of Ti_3_AlC_2_, followed by the formation of 3D crumpled MXene spheres and MXene/ZnO composites via ultrasonic spray pyrolysis. This in situ approach effectively prevented restacking, increased the available surface area, and promoted a more homogeneous ZnO distribution. Bio‐MXene was prepared by in situ HF etching using a LiF/HCl mixture to remove the Al layer from Ta_4_AlC_3_, followed by washing and ultrasonication to obtain few‐layered nanosheets with improved interlayer spacing and reduced restacking [23]. Additionally in the study [24], Ti_3_C_2_T*_x_* is prepared using a dissolution system that relies on in situ generation of HF rather than direct addition. 38% hydrochloric acid is mixed with ammonium fluoride, and Ti_3_AlC_2_ powder is slowly added to this mixture with continuous stirring for several days until the selective dissolution of the aluminum layer is complete. The sample is then washed repeatedly with ethanol and water using centrifugation until the pH reaches approximately 7 and finally dried in an oven at 80.0°C to obtain the final MXene. The reaction of the fluoride with the acid generates HF in situ within the medium, which reduces the risks associated with handling concentrated HF and often results in MXene layers with larger lateral dimensions, fewer defects, and wider interlayer spacing, thus facilitating layer separation and improving the material's quality and properties.

**FIGURE 4:**
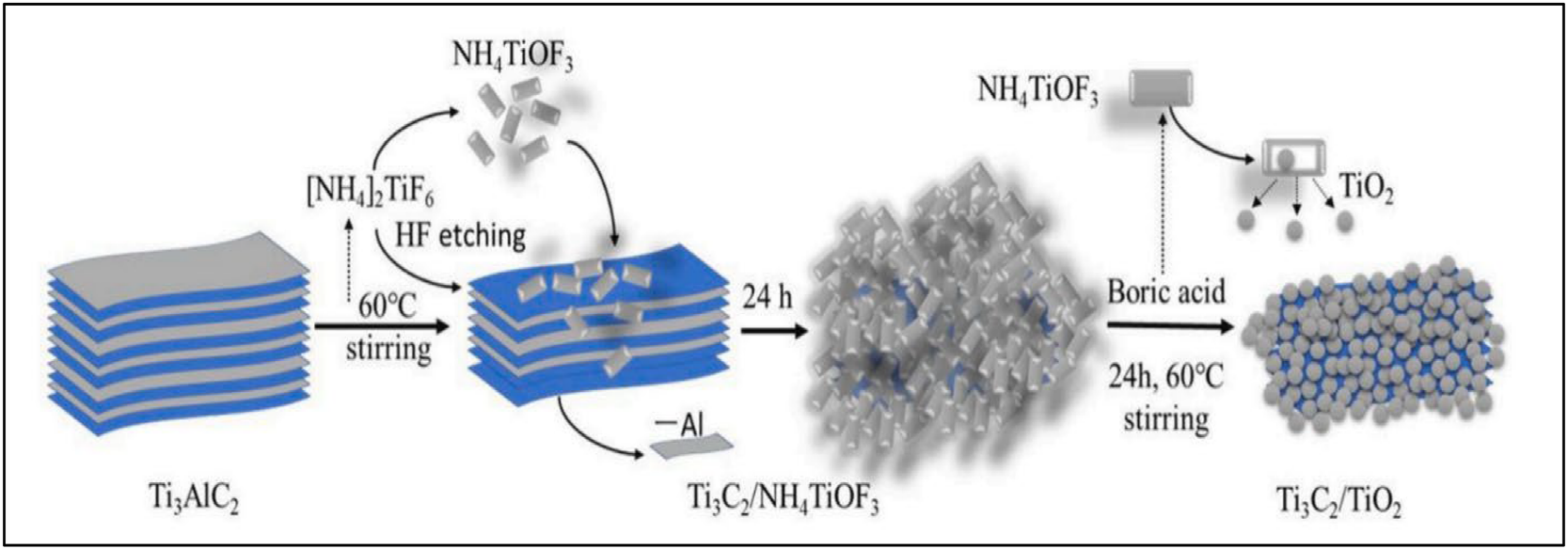
The Ti_3_AlC_2_ exfoliation process and the resulting Ti_3_C_2_/TiO_2_ hybrids. Reproduced from [25] under the CC BY license.

Fluoride salts can be used as multifunctional etching and precursor agents in aqueous media, in addition to traditional HF etching and in situ HF methods based on LiF/NH_4_F with HCl. Such as in a [25] study, ammonium hexafluorotitanate ((NH_4_)_2_TiF_6_) can be used to etch Ti_3_AlC_2_ and supply titanium for oxide development, resulting in Ti_3_C_2_/TiO_2_ hybrids by a single wet‐chemical process as shown in Figure 4. In this system, Ti_3_AlC_2_ is treated with aqueous ((NH_4_)_2_TiF_6_)) at moderate temperature, selectively removing Al and forming Ti_3_C_2_ MXene sheets that become uniformly wrapped by NH_4_TiOF_3_ crystals. A subsequent reaction with H_3_BO_3_ converts NH_4_TiOF_3_ into TiO_2_, yielding Ti_3_C_2_/TiO_2_ structure without the use of bulk liquid HF.

#### 2.2.2 Aqueous Etching Methods without Fluoride

**FIGURE 5:**
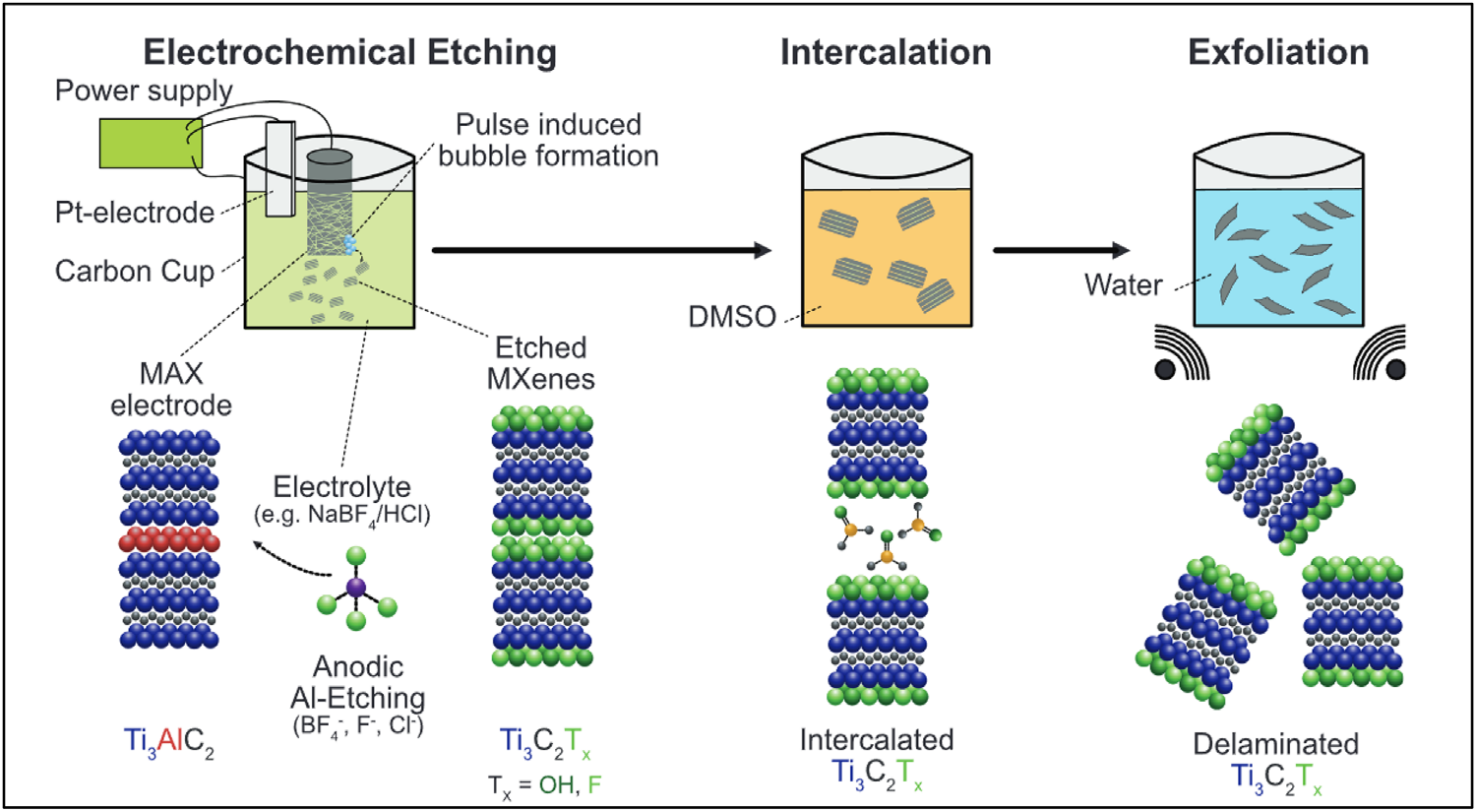
Setup and methodology for electrochemical etching Ti_3_AlC_2_ pellet electrodes in aqueous electrolytes (NaBF_4_/HCl). Electrochemical pulse‐induced bubble formation is used for etching, followed by DMSO intercalation of multilayered MXene and water sonication for delamination into EC‐MXene flakes. Surface termination progression (T*_x_* = —OH, —F, —Cl) from etched multilayered MXene by intercalated (DMSO) stage to delaminated single/few‐layer MXene is depicted in schematics. Reproduced from [28] under the terms of the CC BY‐NC‐ND license.

Despite the efficiency of HF‐based etching procedures in removing the A element from MAX phases, they typically yield F‐terminated MXenes, which can have negative effects on electrical conductivity and create safety issues because of residual HF. In this regard, fluorine‐free etching techniques have received a lot of attention since they provide a safer and more controlled way to MXene production. These approaches allow for improved control over MXene surface chemistry and properties without using fluorine‐containing chemicals [26]. Electrochemical etching in aqueous electrolytes has emerged as a major fluoride‐free strategy for MXene synthesis, resolving many of the environmental and safety problems associated with traditional HF‐based approaches. This technique removes the A‐layer from MAX phases under electrical potentials, eliminating the requirement for harmful fluorinated etchants. Electrochemical etching, as opposed to traditional chemical etching, provides improved sustainability, greater control over morphology and surface chemistry, and the potential to reduce procedure complexity, making it a promising route for the formation of highly efficient MXenes in energy storage and related applications. Electrochemical etching of MXenes involves using the parent MAX phase as an electrode in a three‐electrode cell. An applied potential selectively eliminates the A layer while retaining the transition metal carbide or nitride layers. The weaker M‐A bonds break, and the A atoms dissolve into the electrolyte. Surface terminations such as —O, —OH, and —Cl can be introduced or modified during the process, allowing for precise control over MXene surface chemistry and shape. This approach was applied to various MXene precursors, including Ti‐, Nb‐, V‐, and Mo‐based MAX phases, resulting in fluoride‐free MXenes with improved electrochemical performance. In addition, electrochemical etching has advantages over conventional chemical etching in terms of delamination, adjustable morphology, and sustainability [27]. Recent research indicates possible applications of aqueous electrochemical etching for fluoride‐free MXene production. Pulsed electrochemical exfoliation in aqueous electrolytes, such as HCl and sodium tetrafluoroborate (NaBF_4_)/HCl, can create high‐quality MXene sheets without using dangerous fluorinated etchants as shown in Figure 5. Cathodic pulse voltammetry improves etching efficiency and delamination of MXene layers in situ, resulting in fluoride‐free MXene with superior structural characteristics and purity. This approach enables scalable, green synthesis of MXenes in aqueous conditions while preserving excellent performance and sustainability [28].

Besides electrochemical routes, purely chemical aqueous etching without fluoride has also been reported. An example of a fluoride‐free aqueous etching process is an HCl‐based hydrothermal approach, which uses concentrated hydrochloric acid at elevated temperatures to etch Mo_2_Ga_2_C and produce Mo_2_CTx. Mo_2_CTx with primarily —O/—Cl terminations performs better electrochemically than HF‐etched samples due to the absence of inert —F groups and a more hydrophilic, oxygen‐rich surface. Similar HCl‐hydrothermal conditions have been effectively applied to other MAX phases, demonstrating that powerful nonfluoride acids may efficiently etch MAX phases under careful temperature and reaction time control [29].

Alkali‐assisted hydrothermal etching is another fluoride‐free aqueous method. Potassium hydroxide‐assisted hydrothermal etching was studied to produce fluoride‐free Ti_3_C_2_T*_x_* from Ti_3_AlC_2_, integrating hydroxyl groups directly during one‐pot synthesis. Higher KOH concentrations enhanced etching efficiency, MXene quality, and yield, while yields were still lower than those of fluoride‐based techniques. Thermal pretreatment of the MAX phase 290°C and extended reaction periods up to 64 h at elevated temperatures resulted in full delamination as well as the formation of titanate nanofibers. However, alkali‐etched MXenes have low stability due to quick oxidation of —OH terminations and the difficulties of creating free‐standing films, which can be alleviated by MXene–polymer composites that improve ion transport and inhibit restacking [30].

#### 2.2.3 *Lewis Acidic Etching of Molten Salt* (*Emerging Strategy*)

The conventional top‐down synthesis from MAX phases still relies primarily on concentrated HF or in situ generated fluoride etchants, which are highly corrosive, toxic, and pose serious safety and scalability concerns. To overcome these limitations, Lewis acid molten salt etching was recently invented as an alternative, common, and easily adaptable route to obtain and modify MXenes by treating MAX precursors in halide molten salts. This HF‐free technique not only broadens the variety of MXene compositions available, but it also improves surface termination control and allows for the in situ deposition of metal nanoparticles on MXene surfaces [31]. In one example, Ti_4_AlN_3_ is treated in a LiF–NaF–KF molten mixture (29:12:59 wt%) at 550°C under argon to produce Ti_4_N_3_, and the etching is finished in about 30 min. Ti*_n_* N_*n*−1_ is less stable than Ti*_n_* C_*n*−1_; therefore it dissolves in HF or fluoride‐based acids. This makes the molten salt approach advantageous due to its short processing time. However, rinsing with H_2_SO_4_ and deionized water, followed by delamination in TBAOH, is still required. The resulting Ti_4_N_3_ shows lower crystallinity than MXene produced by HF etching, with TiO_2_ impurities observable in the final product. All things considered, molten salt etching is especially helpful for creating MXenes that are unstable in HF/fluoride‐based acid solutions. However, it has several disadvantages, including the need for a high temperature and consequently higher energy consumption, the fact that the resulting MXene usually has lower crystallinity and purity, and a higher density of vacancies and defects [31, 32].

### 2.3 Bottom‐Up Growth of MXene

#### 2.3.1 CVD Method

Bottom‐up growth is a fundamentally distinct approach to MXene synthesis in which 2D transition metal carbide or nitride layers are formed directly from atomic or molecular precursors, avoiding the requirement for selective etching of a pre‐existing MAX phase (top‐down). This technique has gained popularity in recent years because it allows for the manufacture of MXene compositions that are either unavailable by MAX phase etching or difficult to accomplish with the required purity, crystallinity, and size control. Bottom‐up methods typically provide greater control over layer thickness, lateral dimensions, defect density, and surface terminations, but they frequently have poorer yield, higher equipment needs, and limited scalability as compared to top‐down routes [33]. Xu et al. developed bottom‐up growth of 2D transition‐metal carbides by using chemical vapor deposition (CVD) to synthesize ultrathin Mo_2_C crystals on copper substrates. In this method, highly crystalline carbide layers were grown directly from gaseous precursors at high temperature, completely avoiding the selective etching of MAX phases that are typical of top‐down MXene synthesis. The Mo_2_C nanosheets had uniform thickness at the Nanometer scale and lateral dimensions of several micrometers. This study provided early experimental evidence that CVD‐based bottom‐up routes can achieve precise control over crystallinity, morphology, and structural order, laying the conceptual groundwork for subsequent bottom‐up approaches to MXene‐like materials and related 2D carbides and nitrides [34]. Thin Mo_2_C films and MXene‐like structures can be formed through a PVD‐CVD hybrid technique. Thin copper layers (∼50 nm) placed on molybdenum substrates act as catalysts for CVD development at extreme temperatures 1086–1150°C. Catalyst thickness significantly impacts nucleation density, growth morphology, and surface coverage. Thinner layers achieve ∼86% coverage while limiting undesirable Mo‐Cu alloy formation. Raman spectroscopy, XRD, and EDX analysis revealed good crystallinity and homogeneity. This approach allows for exact control over film characteristics and provides a consistent route for producing high‐quality MXene‐like 2D materials suited for electronic applications [35]. Yorulmaz et al. synthesized ultrathin, pure Mo_2_C MXene flakes on Mo/Cu foils at 1100°C under atmospheric pressure. They used CH_4_ as a carbon source and H_2_/N_2_ carriers to tune thickness (7–150 nm) and coverage (11–68%) via CH_4_ flow, Cu/Mo ratio, and N_2_/H_2_ ratio, avoiding etchant‐induced surface functionalization for superior metallic properties in energy applications. Flakes were applied on SiO_2_/Si via photoresist‐assisted FeCl_3_ etching. Observed problems include high CH_4_ fluxes producing multilayer thickness (due to preferential CH_4_ adsorption on Mo_2_C), pure H_2_ limiting coverage via etching effects, and trace Cl contamination in EDX during transfer [36]. In general, bottom‐up vapor‐phase routes (CVD and PVD‐CVD hybrids) can produce high‐quality Mo_2_C MXene‐like films with tunable thickness and coverage. However, they require higher temperature, more complex equipment, and challenges in large‐scale production compared to traditional top‐down etching routes.

#### 2.3.2 Template‐Assisted Methods

In addition to CVD, template‐assisted methods have been developed for fabricating 2D transition‐metal carbides and nitrides (MXene‐like materials). Compared to CVD techniques, these template pathways can produce much greater yields. This process involves preparing 2D transition‐metal oxide (TMO) nanosheets, which are subsequently carbonized or nitrided to form 2D carbides or nitrides with a structure influenced by the parent TMO [37]. For instance, Xiao et al. demonstrated that oxide templates can guide the production of 2D nitride nanosheets by reporting a scalable method that reduces 2D hexagonal oxides in ammonia to produce subnanometer‐thick 2D MoN nanosheets. The resulting MoN nanosheets exhibited a uniform morphology, excellent dispersibility in water as evidenced by the Tyndall effect, and an ultrathin thickness of approximately 0.71 nm, confirming successful template‐directed formation of well‐defined 2D nitride nanosheets [38]. Jia et al. developed a template‐assisted method for manufacturing ultrathin N‐doped Mo_2_C nanosheets by first preparing MoO_2_ nanosheets via chemical vapor reduction of MoO_3_ in Ar/H_2_ at 900°C and then converting them with dicyandiamide at 450 and 700°C. The MoO_2_ nanosheets served as a 2D template, allowing for the retention of nanosheet morphology in the final product [39].

**FIGURE 6:**
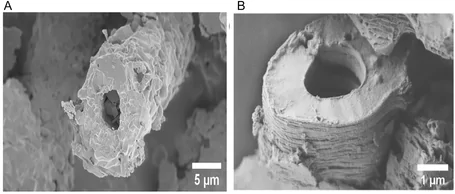
(A) SEM of Ti_3_AlC_2_ MAX phase produced at 1250°C with a 10 h dwell period by molten salts in air. (B) SEM of tubular Ti_3_C_2_T*_x_* MXene produced at 700°C with a 40 min dwell period by molten salts in air. Reproduced from [40] under the terms of the Creative Commons Attribution 4.0 International License (CC BY 4.0).

Oliveira et al. used carbon fibers (CFs) as a carbon source and morphological template in molten salt shielded synthesis (MS^3^) to create tubular Ti_3_Al_1.__2_C_1.__8_ MAX phases at 1250°C for 10 h. The Ti_3_C_2_T*_x_* MXene produced through selective etching retains its unique tubular form with aligned 2D layers along the carbon fiber scaffold. This method reveals how template engineering can impart anisotropic structures to MXenes, improving carrier transport and offering applications in high‐rate Li‐ion battery anodes, gas sensing, and membrane filtration. SEM micrographs confirm the efficiency of molten salt shielded synthesis (MS^3^). After 10 h at 1250°C, the nonstoichiometric Ti_3_Al_1.__2_C_1.__8_ MAX phase forms homogenous tubular microrods with sizes ranging from 2 to 5 μm (Figure 6A). Compared to the 7 h sample, the extended reaction period results in better morphological homogeneity, indicating a more complete response around the carbon fiber scaffold. After selective etching of Al, Figure 6B shows that the Ti_3_C_2_T*_x_* MXene retains the tubular topology, with smooth and well‐aligned 2D layers. The retention of morphology during the MAX‐to‐MXene transformation demonstrates the structural stability of this template‐directed method. Such anisotropic tubular shapes can improve charge and ion transport, making them suitable for high‐rate battery electrodes and, perhaps, filtering applications [40].

### 2.4 Influence of Synthesis Conditions and Surface Functionalization on MXene Properties

MXene synthesis is closely associated with delamination, intercalation, and surface terminations, which together determine the final structural and functional properties of the material. In particular, the choice of etching route influences how effectively the A‐layer is removed from the MAX phase, while subsequent intercalation and delamination steps expand the interlayer spacing and expose more active sites. These post‐etching processes also affect the dominant surface terminations, such as —F, —OH, and —O, which play an important role in tuning hydrophilicity, conductivity, and ion transport behavior [41]. Oxygen‐containing terminations have been widely reported to promote electrical conductivity, whereas hydroxyl groups improve hydrophilicity by hydrogen bonds. Fluorine terminations can add to environmental stability, although excessive —F coverage can passivate active sites and impair electrochemical performance. The nature and distribution of these surface terminations are strongly linked to the synthesis technique and experimental conditions [5]. The synthesis method and experimental setup have a significant impact on the type and distribution of these surface terminations. According to Mashangva et al.'s [42] study, the one‐step microwave‐assisted method was superior to the traditional HF etching route because it greatly shortened the synthesis time, allowed for delamination without the need for intercalation compounds, and enhanced the electrochemical performance in supercapacitor applications. The various synthesis techniques used for MXene preparation are listed in Table 1, which compares the most used MXene synthesis pathways in terms of yield, scalability, safety, defect density, and termination control. This comparison illustrates the literature regularly demonstrates that increases in one parameter frequently come at the expense of another. Furthermore, the functional groups on the MXene surface promote efficient charge transfer and redox reactions, making MXenes highly suitable for use in batteries, supercapacitors, and electrocatalytic systems [46]. For example, the —O functional group can behave as an active site for the hydrogen evolution reaction (HER), whereas a high —F surface coverage correlates with increased electrical conductivity. As a result, altering the kind and proportion of functional groups is a useful technique for increasing electrocatalytic performance [43]. In this context, HF‐etched Ti_3_C_2_T*_x_* is enriched in —F groups relative to —O and —OH, while the HCl–LiF route yields mainly oxygen‐containing surface terminations. Furthermore, the —F coverage on HF‐etched Ti_3_C_2_T*_x_* is about 4 times higher than that of the HCl–LiF‐derived material [45].

**TABLE 1:** Comparative assessment of MXene synthesis routes.

Route	Yield	Scalability	Safety/environmental impact	Termination control	Structural quality/defects	Main advantage	Main limitation	Ref.
**HF etching**	High etching efficiency in optimized conditions	Moderate for lab‐scale; limited industrially	Severe safety and environmental concerns	Good, but often F^–^/O^–^/OH^–^ rich terminations	Good crystallinity in optimized conditions	Most established and widely used	Severe safety risks and hazardous waste	[43, 44]
**In situ HF**	High to moderate–high	Better than direct HF, still batch‐dependent	Improved handling safety, but still fluoride‐based	Termination ratio depends on etching parameters.	Often larger flakes and fewer defects than direct HF	Safer, more controllable than direct HF and expands the interlayer spacing in a single procedure.	Still relies on fluoride chemistry	[44, 45]
**Electrochemical etching**	Limited or developing yield	Promising, but still emerging	Very good; fluoride‐free and greener	Cleaner surface chemistry and tunable termination	Good purity/lower contamination	Sustainable and tunable	Process maturity and throughput still developing	[26, 27, 46]
**Hydrothermal alkali etching**	Moderate to low	Limited; reaction time often long	Good; fluoride‐free	Good for OH‐rich surfaces	May suffer from oxidation/lower stability	Directly introduces hydroxyl terminations	Lower yield and stability issues	[5, 26]
**Molten‐salt Lewis acidic etching**	Moderate	Promising for scale‐up, but temperature‐intensive	Better than HF; HF‐free	Good to moderate, composition‐ dependent	Defect density depends on synthesis conditions	HF‐free and composition‐expanding	High temperature and defect formation	[26]
**Bottom‐up/CVD**	Low for bulk production	Poor for mass scale; excellent for precision films	Good; no HF etching	Excellent atomic‐level control	high crystallinity, low defects	Best for high‐quality, tailored films	Expensive, complex, and low throughput	[26, 47]

### 2.5 Postsynthesis Processing for MXene‐Based Nanostructures

#### 2.5.1 *Solvothermal* *and Microwave‐Assisted Techniques*

**FIGURE 7:**
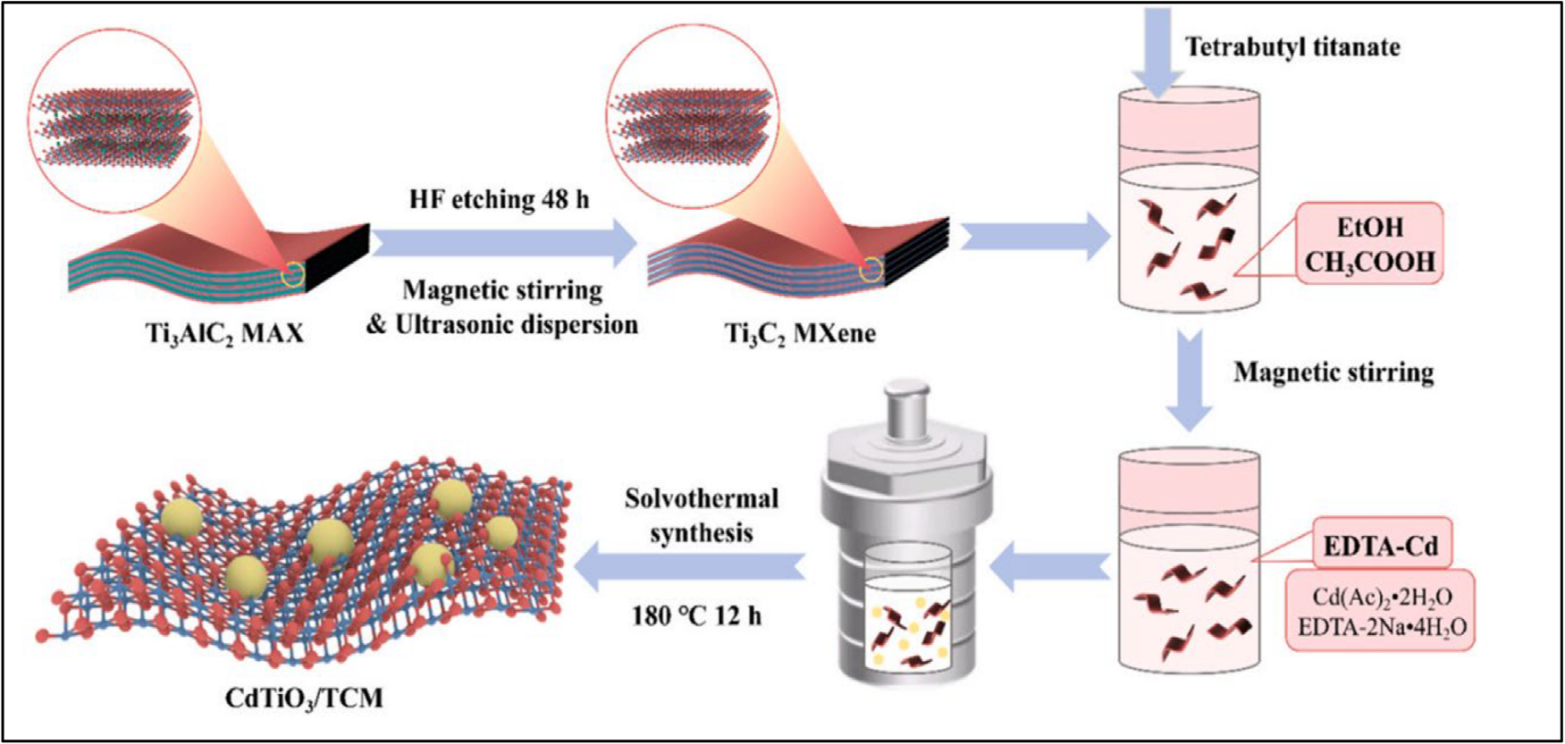
Illustration of the coordination‐solvothermal synthesis process for CdTiO_3_/Ti_3_C_2_ MXene heterojunctions. Reproduced with permission [48] Copyright 2024, Elsevier (License No. 6264480266654).

Solvothermal synthesis is a versatile approach for growing semiconductor nanoparticles directly on MXene sheets. This allows for strong interfacial contact and controlled morphology in MXene‐based heterojunctions. As a representative example, Li et al. employed a coordination‐solvothermal route to anchor shuttle‐like CdTiO_3_ nanoparticles onto multilayer Ti_3_C_2_ MXene sheets, forming CdTiO_3_/TCM Schottky heterojunctions. Multilayer Ti_3_C_2_ was first obtained by HF etching of Ti_3_AlC_2_, and then CdTiO_3_ grew in situ from an EDTA–Cd precursor and tetrabutyl titanate in an ethanol‐based solvothermal system at 180°C, as shown in Figure 7. This process yielded tightly coupled CdTiO_3_/Ti_3_C_2_ interfaces with increased BET surface area and enhanced visible‐light absorption compared with bare CdTiO_3_. As a result, the optimized CdTiO_3_/TCM‐B sample exhibited a RhB degradation rate constant about 7 times higher than pristine CdTiO_3_, highlighting how solvothermal growth on MXene can engineer efficient charge‐transfer pathways in MXene‐based photocatalysts [48]. In addition to from semiconductor–MXene heterojunctions, solvothermal techniques have been widely used to develop 3D MOF structures directly on MXene sheets in one‐ or two‐pot systems [49]. Under solvothermal circumstances, MOF crystals like MIL‐53‐NH_2_ grow in situ on delaminated Ti_3_C_2_T_*x*_ to generate MXene@MIL‐53‐NH_2_ hybrids with uniformly dispersed 3D frameworks anchored onto the 2D MXene backbone. In contrast with naked MXene, this controlled interfacial growth maintains the distinctive spindle‐like morphology of MIL‐53‐NH_2_ while guaranteeing close contact with MXene. This reduces nanosheet restacking and greatly expands the accessible surface area and number of adsorption–reduction active sites [50].

In addition to traditional solvothermal methods, microwave‐assisted approaches have become a quick and energy‐efficient way to modify the interfaces and structure of MXene‐based nanocomposites. Microwave‐assisted synthesis not only shortened the preparation time but also promoted uniform La incorporation, improved crystallinity, and enhanced surface properties of the La/Ti_3_C_2_T_*x*_ composite [51]. Ti_3_C_2_ MXene was first produced from Ti_3_AlC_2_ by etching and delamination, and then it was added in controlled loadings during a microwave‐assisted synthesis of TiO_2_. This resulted in MXene–TiO_2_ nanocomposites with high crystallinity, tuned phase composition, and increased specific surface area, which significantly improved the removal of amoxicillin and methylene blue when compared to pristine TiO_2_ [52]. For advanced electrochemical applications, microwave‐assisted procedures have also been used as quick ways to synthesize and then modify MXenes. Ti_3_C_2_T_*x*_ MXene was created in one study by HF‐based etching of Ti_3_AlC_2_ under microwave irradiation in a matter of minutes. It was then mixed with hydrothermally generated V_2_O_5_ via a straightforward ultrasonication procedure to create Ti_3_C_2_T_*x*_@V_2_O_5_ nanocomposites. When compared to pure Ti_3_C_2_T_*x*_, the resultant hybrids showed significantly improved specific capacitance and cycling stability. This was because the highly conductive MXene scaffold promoted ion diffusion and charge transport, while V_2_O_5_ offered more redox‐active intercalation sites for charge storage [19].

#### 2.5.2 *Calcination*, *Coprecipitation*, *and Sol–Gel for MXene‐Based Composites*

Calcination, coprecipitation, and sol–gel processing are commonly utilized as postsynthesis methods to fabricate MXene‐based composites, wherein pre‐etched MXene functions as a conductive scaffold for the regulated creation or alteration of secondary phases [53]. Some studies illustrate how post‐synthesis wet‐chemical and thermal routes can be combined. For example, Shafique et al. reported a Ti_3_C_2_T_*x*_/CuCr_2_O_4_ nanocomposite in which CuCr_2_O_4_ spinel was initially produced via a citrate‐assisted sol–gel method, turning aqueous Cu^2+^/Cr^3+^ precursors into a polymeric gel that was subsequently calcined at 750°C to yield phase‐pure CuCr_2_O_4_ nanoparticles. CuCr_2_O_4_ was redispersed in an ethylene glycol/acetic acid medium after pre‐etched TiC_2_T_*x*_ MXene was dispersed in water. Sonication and strong stirring of these suspensions produced a coprecipitation‐type assembly that produced a sandwich‐like MXene/CuCr_2_O_4_ architecture. This hierarchical structure produced a specific capacitance of roughly 445 F g^−1^ in 0.1 M H_2_SO_4_ with good stability over 500 cycles [54]. For instance, Infancy et al. prepared Ni‐doped cobalt ferrite/MXene nanocomposites (Co_1–*x*_Ni_*x*_Fe_2_O_4_/MXene) by a low‐temperature coprecipitation technique, where Co_1–*x*_Ni_*x*_Fe_2_O_4_ nanoparticles were first precipitated in alkaline medium then combined with delaminated Ti_3_C_2_T_*x*_ MXene by ultrasonication and drying to form well‐distributed ferrite/MXene hybrids. Increasing Ni content in Co_1–*x*_Ni_*x*_Fe_2_O_4_ reduces crystallite size and lattice parameter. Integration with Ti_3_C_2_T_*x*_ MXene increases surface area and specific capacitance (up to ∼ 520 F g^−1^ at 10 mV s^−1^), demonstrating how coprecipitation‐derived ferrites can be effectively combined with MXene to engineer high‐performance supercapacitor electrodes [55]. Combining wet‐chemical coprecipitation with heating or assembly procedures offers a flexible postsynthesis method for adjusting and optimizing MXene‐based composites’ performance.

#### 2.5.3 Ball‐Milling and Other Mechanical Methods

**FIGURE 8:**
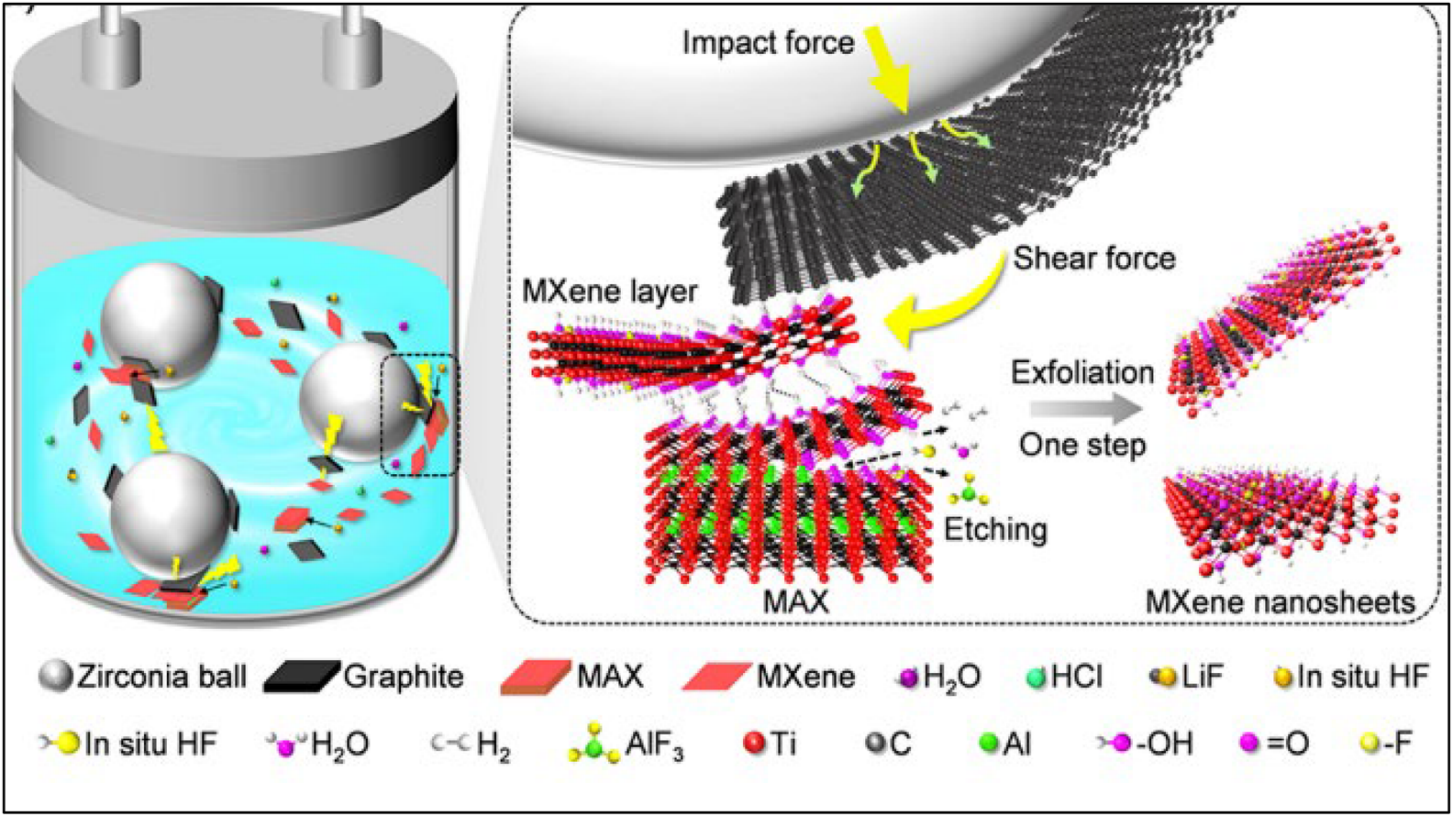
A one‐step graphite‐assisted ball‐milling (GABM) method that simultaneously etches the MAX phase and mechanically exfoliates MXene nanosheets. Reproduced from [21] CCS Chemistry Open Access.

MXene can also be produced using mechanochemical synthesis, particularly high‐energy ball milling. This technique supports the subsequent etching and exfoliation processes or aids in the removal of the A‐layer from MAX stages by applying mechanical forces. Ball milling can impact MXenes’ electrochemical performance by increasing interlayer spacing, promoting delamination, and improving ion accessibility. Additionally, one‐step exfoliation procedures and fluorine‐free mechanochemical methods that demonstrate scalable and solvent‐minimized manufacturing pathways have been reported. Despite advantages like scalability and ease of usage, issues including contamination, structural defects, and possible amorphization persist [56]. Several mechanical techniques have been investigated for MXene preparation and size manipulation in addition to traditional high‐energy ball milling [56]. By reducing the size of MAX or MXene particles and encouraging solid‐state interactions, mechanical grinding or attrition milling can facilitate surface modification or mechanochemical etching [57]. On a relatively large scale, mechanical exfoliation in a liquid environment has also been used to delaminate layered precursors into few‐layer MXene nanosheets [58]. More recently, scalable methods to produce MXenes and their derivatives have been developed using solvent‐free mechanochemical techniques that combine ball milling with solid etchants or dopant sources [56]. MXene nanosheets were recently manufactured using a synergistic graphite‐assisted ball milling (GABM) technique that combined mechanical exfoliation and chemical etching in a single step as shown in Figure 8. This process involves mixing Ti_3_AlC_2_ MAX powder with graphite flakes, zirconia balls, and a LiF/HCl etching solution, followed by vacuum ball milling at room temperature. In situ HF from LiF/HCl solution selectively etches the Al layers in Ti_3_AlC_2_, while shear forces produced during ball milling exfoliate the newly created MXene layers into nanosheets. Graphite serves a dual purpose, buffering the usual impact of the hard zirconia balls, limiting excessive fragmentation, and efficiently transmitting shear pressures to enable lateral exfoliation of large‐area sheets. This GABM technique produces single‐layer Ti_3_C_2_T_*x*_, Ti_2_CT_*x*_, and Ti_3_CNT_*x*_ MXene nanosheets with high yields of 95–97  wt%, (Young's modulus ≈ 0.37  TPa), high electrical conductivity, and strong solution processability. These high‐quality nanosheets can be assembled into continuous fibers, large‐area films, and 3D bulk architectures, enabling MXene films with excellent EMI shielding performance and low midinfrared emissivity, and thus highlighting the potential of ball‐milling‐assisted strategies for scalable MXene production [21].

Most MXene synthesis techniques use high‐energy ball milling as a post‐treatment step after etching the MAX phase to produce Ti_2_CT_*x*_ MXene. The study [59] utilized high‐energy ball milling in air to oxidize Ti_2_CT_*x*_ and produce TiO_2_@C nanosheets. Milling delaminates stacked Ti_2_CT*_x_* by breaking interlayer van der Waals contacts. This results in single‐ or few‐layered nanosheets with lower thickness and shorter oxygen diffusion paths. Ti_2_CT_*x*_ is converted into a 2D TiO_2_@C composite via a straightforward, scalable, and solvent‐free solid‐state process. Mechanochemical approaches provide solvent‐free and scalable MXene synthesis. Before being widely used in industry, structural damage, pollution, and surface termination control issues still need to be thoroughly investigated.

#### 2.5.4 ALD and Electrochemical Modification for MXene Films and Heterostructures

Atomic layer deposition (ALD) and electrochemical modification are effective postsynthesis methods for engineering MXene films and heterostructures beyond stacked nanosheets [60]. This general idea is demonstrated by thermoelectric ZnO@Ti_3_C_2_T_*x*_ composite films, which use low‐temperature ALD to grow homogeneous ZnO layers over laminar Ti_3_C_2_T_*x*_ membranes while maintaining the flexibility and apparent shape of the underlying MXene film. Strong phonon‐interface scattering at the oxide–MXene interfaces suppresses heat transport and reduces the thermal conductivity by about a factor of four, greatly improving the overall thermoelectric performance. At the ZnO/Ti_3_C_2_T_*x*_ interface, a Schottky barrier introduces an energy‐filtering effect that selectively transmits high‐energy carriers, significantly enhancing the Seebeck coefficient and yielding more than a twofold increase in power factor when compared to pristine Ti_3_C_2_T_*x*_ [61]. Beyond oxide coatings grown by ALD, electrochemical modification provides a complementary post‐synthesis route to engineer MXene films. The available interlayer space and active site density of MXene electrode materials can be greatly increased by postsynthesis electrochemical and surface modification techniques. For instance, compared to pure MXene, a NaOH hydrothermal treatment using citric acid as a reducing agent increased the interlayer spacing and changed —F terminations to O‐containing groups, resulting in an ∼250% increase in gravimetric capacitance 543 F g^−1^ at 2 mV s^−1^. Increased active surface sites and improved electrolyte ion accessibility are responsible for this improvement [62].

## 3 MXenes for Catalysts

### 3.1 MXenes for Photocatalysis

The catalytic use of light energy has been extensively researched as a means of solving modern difficulties in energy demand, resource depletion, and environmental contamination, particularly in the chemical and process industries. Photocatalytic processes turn solar energy into chemical reactions, eliminating the need for fossil‐fuel‐based heat or power. This makes them a viable option for long‐term energy production and pollution reduction [63]. Photocatalytic oxidation is a green and adaptable method that uses sunlight or low‐energy artificial light to mineralize a variety of organic contaminants at ambient temperature and pressure [64]. A recent study highlighted the relevance of MXene‐based catalysts in pharmaceutical wastewater cleanup by demonstrating the increased visible‐light photocatalytic degradation of cetirizine using V_2_O_2_‐modified MXene nanocomposites [65]. Photocatalysts in wastewater treatment and air purification generate reactive oxygen species (ROS) (such as hydroxyl and superoxide radicals) that attack and decompose organic contaminants into less harmful inorganic products like CO_2_ and H_2_O. This process is suitable for continuous environmental remediation applications [64].

In recent years, MXene‐based composites have been promising co‐photocatalysts due to their high electronic conductivity, tunable surface terminations, and large specific surface area. These properties facilitate charge separation and electron transfer at the catalyst interface [66]. MXene‐based photocatalysts are typically produced utilizing three methods: mechanical mixing, self‐assembly, and in situ decoration/oxidation. Mechanical mixing is the most straightforward method, which typically includes spreading the MXene and semiconductor photocatalyst in a liquid medium (by stirring or sonication) or physically grinding their powders to form a composite. Self‐assembly, on the other hand, relies on electrostatic interactions, in which negatively charged MXene sheets spontaneously join with positively charged photocatalysts, resulting in well‐organized photocatalyst composites [67]. MXene‐based photocatalysts have been merged with a wide range of semiconductors, such as metal oxides (TiO_2_, Nb_2_O_5_), metal sulfides (CdS, ZnS, Zn_2_In_2_S_5_, CdLa_2_S_4_), polymeric photocatalysts (g‐C_3_N_4_), and MOFs or dye‐sensitized systems. These MXene‐containing heterostructures have significantly improved photocatalytic activities in H_2_ evolution, CO_2_ reduction, organic pollutant degradation, and N_2_ fixation. This is due to Schottky junction formation, accelerated charge separation, increased surface area, and abundant surface functional groups which provide active redox sites [68]. TiO_2_ is a popular metal oxide to combine with MXene due to its low toxicity, strong photocatalytic activity, photochemical stability, and chemical robustness [66, 69, 70]. The combination of TiO_2_ with MXene has been shown highly efficient in overcoming the inherent limits of pristine TiO_2_ by decreasing the effective band gap, inhibiting electron–hole recombination, and improving the adsorption and degradation of organic contaminants [67]. This makes MXene/TiO_2_ heterostructures a promising platform for photocatalytic degradation and solar‐driven environmental applications [25].

**FIGURE 9:**
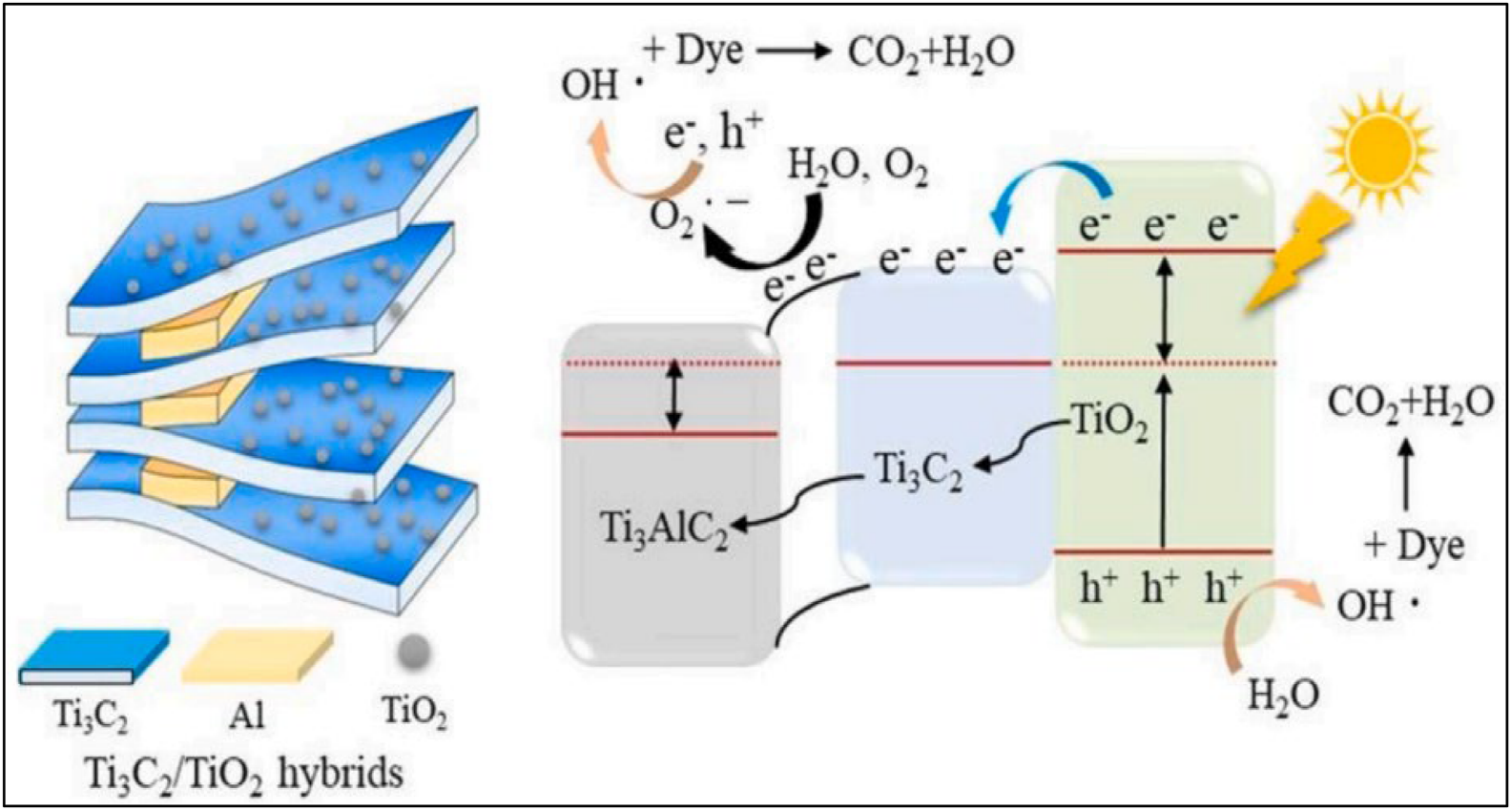
Illustrates the mechanism of photocatalysis of methylene blue (MB) on a Ti_3_C_2_/TiO_2_ heterostructure. Reproduced from [25] under the CC BY license.

Figure 9 shows that photocatalytic degradation of Ti_3_C_2_/TiO_2_ MXene‐based hybrids follows a normal semiconductor‐mediated radical pathway. The MXene phase acts as an efficient electron sink, suppressing charge recombination. When exposed to high‐energy photons, electrons in the valence band of anatase TiO_2_ are stimulated to the conduction band, resulting in positively charged holes. Photogenerated electrons move quickly to Ti_3_C_2_ (and leftover Ti_3_AlC_2_) domains due to their reduced work function. This allows Ti_3_C_2_ to act as an electron reservoir and extend the lifespan of charge carriers. Electrons on the Ti_3_C_2_ surface react with dissolved oxygen to produce superoxide radicals (O_2_^−^), whereas holes in the TiO_2_ valence band oxidize surface‐adsorbed hydroxide or water molecules to make hydroxyl radicals (—OH). Both radical species have a high oxidizing power and target the adsorbed organic dye (e.g., methylene blue), gradually breaking down its chromophore structure and mineralizing it into simpler oxidation products such as tiny inorganic ions and carbon‐based species. The NH_4_TiOF_3_ precursor produces nanoparticulate anatase TiO_2_, which enhances the photocatalytic effectiveness of Ti_3_C_2_/TiO_2_ hybrids by increasing surface roughness and dye adsorption capacity [25]. MXene has been effectively coupled with a wide range of semiconductor photocatalysts, including BiVO_4_, Ag_3_PO_4_, In_2_S_3_, CdS, BiFeO_3_, BiOBr, g‐C_3_N_4_, AgInS_2_, and MIL‐100(Fe) to produce efficient MXene‐based heterostructures for different photocatalytic processes. These MXene‐based nanocomposites have shown outstanding performance in photocatalytic degradation of dyes and phenolic pollutants, CO_2_ reduction to useful fuels (CH_4_, CH_3_OH, C_2_H_5_OH), H_2_ production from water splitting, and N_2_ fixation to NH_3_ or NO_3_^–^, mainly due to their optimized band alignment, enhanced charge‐carrier separation, multiple surface reactive sites, and the dominant participation of ·O_2_^–^, ·OH, and h^+^ species in the overall reaction pathways [67].

### 3.2 MXenes for Electrocatalysis

Electrocatalysis plays a crucial role in clean energy technologies such as water electrolyzers, fuel cells, CO_2_ electrolyzers, and nitrogen reduction devices. Efficient and long‐lasting electrocatalysts are required for successful operation. Modern catalysts often use metallic nanoparticles spread on conductive substrates for a large surface area, electrical conductivity, and stability under harsh electrochemical conditions. Carbon materials including graphene, nanotubes, and nanofibers are commonly used as benchmark supports. However, their poor electrochemical endurance and weak metal–support interactions might cause particle separation, migration, and coalescence during long‐term use [71]. Alternative support materials, such as metal oxides, nitrides, carbides, and developing 2D systems, are being investigated to improve stability and strengthen metal–support interactions in advanced electrocatalytic composites. Because of their hydrophilic surfaces, chemically adjustable terminations, and metallic conductivity, 2D MXenes have become attractive platforms for sustainable electrocatalysis [72]. As a result, MXene‐based catalysts have exhibited outstanding performance in HER and increasingly competitive activity in oxygen evolution reaction (OER) and oxygen reduction reaction (ORR), particularly when incorporated into heterostructures or composite systems [73]. This prominence is reflected in research trends among MXene‐based electrocatalytic studies, where hydrogen and oxygen evolution reactions (HER and OER) are the most extensively studied topics, accounting for around 28.7% and 10.4% of reported works, respectively [74].

#### 3.2.1 HER

One of the key challenges in contemporary electrochemical energy conversion is the rational development of highly efficient and economical electrocatalysts for the hydrogen evolution process (HER). MXenes have garnered significant attention lately because of their high electrical conductivity and chemically customizable surfaces, which set them apart from traditional transition‐metal combinations. In addition to facilitating quick electron transit, their layered architecture allows for synergistic coupling in composite systems and heterostructures. As a result, MXene‐based materials are becoming more widely acknowledged as flexible platforms for HER electrocatalysis of the future generation [72]. While Pt/C is still the HER benchmark due to its superior kinetics, its exorbitant cost, unavailability, and carbon support corrosion severely limit practical scaling. MXenes solve these problems by providing high metallic conductivity, strong Ti–C frameworks, and stable —F terminations that inhibit nanoparticle aggregation and offer greater durability under operational settings [75]. Pristine Ti_3_C_2_T_*x*_ MXene has intrinsic electrocatalytic activity for the HER. The HF etching of Ti_3_AlC_2_ MAX phase yields multilayered Ti_3_C_2_T_*x*_ MXene with a low overpotential of ∼147 mV at 10 mA cm^−2^ and a Tafel slope of ∼ 80 mV dec^−1^. Ti_3_C_2_T_*x*_ outperforms Pt/C in HER activity across 50 h and 5000 cycles, indicating its stability and longevity in acidic environments. The presence of —O and —F surface terminations, as well as the layered architecture, all contribute considerably to increasing HER activity [76]. To improve catalytic performance, MXenes can be hybridized with transition metal compounds, such as sulfides, phosphides, or oxides. For example, the hybridization of MXenes (Ti_3_C_2_T_*x*_) with transition metal sulfides (CoS_2_) improves HER performance in alkaline media. The Ti_3_C_2_T_*x*_/CoS_2_ electrocatalyst has a low overpotential of 286 mV for 20 mAcm^−2^, a Tafel slope of 78 mV dec^−1^, a large electrochemical surface area of about 495 cm^2^, and a low charge transfer resistance (*R*_ct_) of 115.0 Ω. The synergy between the highly conductive 2D MXene structure and the catalytic activity of CoS_2_ leads to enhanced electron transport, better ion accessibility, and favorable Volmer–Heyrovsky kinetics. These MXene‐based hybrids show the possibility for strong, non‐noble‐metal HER electrocatalysts for practical hydrogen production applications [77].

#### 3.2.2 OER

OER is especially important in clean‐energy technologies such as total water splitting and metal‐air batteries, where the slow multistep oxygen evolution process remains a significant kinetic bottleneck. Therefore, creating cost‐effective and reliable OER electrocatalysts is critical for enabling scale hydrogen production and sustainable energy conversion systems [78]. MXenes are becoming increasingly popular for the oxygen evolution process (OER) due to their metallic‐like conductivity, layered structure, and customizable surface chemistry. Their high electrical conductivity allows for efficient electron transport and lowers charge‐transfer resistance under anodic circumstances, boosting OER kinetics. MXenes’ hydrophilic surfaces, often ending with ‐O, ‐OH, and ‐F groups, facilitate excellent dispersion in aqueous electrolytes and improve interfacial contact with water molecules. These surface terminations also alter the adsorption behavior of oxygen‐containing intermediates, which is important for modifying catalytic activity. Furthermore, MXenes’ electronic structure and catalytic characteristics can be adjusted using transition metal selection, surface termination engineering, and interlayer spacing control, allowing for optimal active site exposure and adsorption energetics. Several Ti‐, Mo‐, Nb‐, V‐, and Cr‐based MXenes have been investigated for water splitting applications. In addition to being intrinsic electrocatalysts, MXenes work well as conductive platforms for creating heterostructures with metal oxides, phosphides, or sulfides, where synergistic effects improve activity and stability. However, their sensitivity to oxidation in aqueous conditions remains a barrier, encouraging ongoing research into structural and surface stabilizing techniques [79]. The number of MXene‐based OER publications have increased significantly, from fewer than ten in 2018 to around 130 in 2024 and more than 100 in 2025, indicating a growing interest in this topic [74]. Early research revealed that pristine MXenes have negligible intrinsic activity for oxygen catalysis, so the focus has shifted to hybrid architectures in which MXenes are combined with OER‐active components (e.g., transition‐metal oxides, layered double hydroxides, and metal phosphides/nitrides/sulfides) to achieve synergistic enhancement [80]. For instance, Ti_3_C_2_T_*x*_/NiFe_2_O_4_ nanocomposites have shown a low OER overpotential of 181.0 mV at 10 mAcm^−2^, which is explained by the ferrite nanoparticles and MXene substrate's strong interfacial electronic contacts [81].

#### 3.2.3 Multifunctional Applications

Considerable effort has been made to generate non‐noble metal electrocatalysts that are conductive, cost‐effective, long‐lasting, and capable of demonstrating bifunctional activity for both HER and OER. Bifunctional catalysts have an attraction for overall water splitting systems because they simplify device design and save material prices. However, establishing balanced catalytic performance for both reactions is still difficult due to their different reaction mechanisms and active site requirements [82]. To overcome these limitations, a wide range of substances such as carbides, phosphides, metal oxides, nitrides, and transition metal dichalcogenides have been widely investigated. MXenes, one of these promising options, has recently received more attention. MXenes’ metallic conductivity, redox‐active transition metal centers, and variable surface functions make them suitable for a variety of electrocatalytic processes. These features have made them attractive candidates for HER, ORR, and OER [83]. MXenes’ electrocatalytic activity is mostly determined by their surface terminations, where —F and —O groups can restrict ion transport and modify adsorption energetics. To address these constraints, surface engineering procedures like doping or intercalation with rare‐earth metals have been used to promote charge transfer, reduce unwanted terminations, and create new active sites, resulting in enhanced HER and OER performance [84]. Holey Ti_3_C_2_T_*x*_ MXene nanosheets closely connected with ultrathin Ni–Fe layered double hydroxides (NiFe‐LDH/H‐Ti_3_C_2_T_*x*_), which exhibit remarkable bifunctional HER/OER performance, are an effective example of such surface engineering. A complex 3D nanoarchitecture is produced by the deliberate insertion of in‐plane holes, which shortens ion diffusion pathways, reveals many catalytically active sites, and sustains high charge transfer rates. This design highlights the potential of holey MXene platforms for hosting improved electrocatalysts by performing significantly better than both pure TiO_2_ and bare Ni–Fe LDHs [85]. However, developing efficient bifunctional or trifunctional performance in a single MXene‐based system remains difficult because different electrochemical processes require different active sites and reaction routes. As a result, major efforts have been made to construct surface‐engineered or heterostructured MXenes capable of driving many reactions at once, including HER, OER, and ORR [86]. Table 2 highlights representative studies on MXene‐based multifunctional electrocatalysts and their electrochemical performance.

**TABLE 2:** Electrochemical performance of advanced MXene‐based multifunctional electrocatalyst.

MXene/Hybrid		Functions	Medium	Overpotential			Tafel slope, mV dec^−1^			Stability	Ref.
				HER, mV, at 10 mA cm^−2^	OER, mV, at 10 mA cm^−2^	ORR, mV	HER	OER	ORR		
Ti_3_C_2_T_*x*_/MIL‐53(Fe)/ZIF‐67 (M3)		Bifunctional (HER + OER)	Alkaline	307	237	—	185	64	—	12 h	[87]
Er@Ti_3_C_2_T_*x*_		Bifunctional (HER + OER)	1.0 M KOH	256	381	—	102	157	—	Stable	[84]
V_2_CT_*x*_‐based Pt/Rh single atoms, DFT	Rh‐v‐V_2_CO_2_	Bifunctional (HER + OER)	Theoretical study (DFT, CHE model, aqueous environment)	190	370	—	—	—	—	Stable at 300 K for 5 picoseconds (AIMD simulations)	[86]
	Pt‐v‐V_2_CCl_2_	Bifunctional (OER + ORR)		—	490	550	—	—	—		
	Pt‐v‐V_2_CS_2_	Bifunctional (OER + ORR)		—	580	400	—	—	—		
	Pt‐v‐V_2_CO_2_	Trifunctional (HER + OER + ORR)		80	360	380	—	—	—		
NiCo_2_O_4_/ Ti_3_C_2_T_*x*_		Bifunctional (OER + ORR)	OER: 1.0 M KOH ORR: 0.1 M KOH	—	360	*E*_½_ = 720, *E*_onset_ = 920	—	64	130	97%@10 h OER, 90%@10 h ORR	[88]
CoP@C/Ti_3_C_2_T_*x*_ (CPMX‐360)		Trifunctional (HER + OER + ORR)	OER/ORR: 1.0 M KOH HER: 0.5 M H_2_SO_4_	220	235	*E*_½_ = 740, *E*_onset_ = 820	206	54	—	Stable after 500 CV cycles (HER/OER)	[89]
Fe_MC_–Mo_2_TiC_2_T_*x*_/Graphene (Fe_MC_–MXene/GrH)		Trifunctional (HER + OER + ORR)	1.0 M KOH	142	296	*E*_½_ = 960, *E*_onset_ = 1000	142.8	58.2	69.04	HER 80%@50 h; OER 95%@50 h; ORR 96.2%@60 h + 10 000 CV cycles	[90]
MWCNT/V_2_CT_*x*_ (M2)		Bifunctional (HER + OER)	1.0 M KOH	27	469	—	44	77	—	16 h + 1000 cycles	[91]
Pt‐W_1_._33_C		Bifunctional (HER + ORR)	HER: 0.5 M H_2_SO_4_ ORR: 0.1 M KOH	36	—	320 at 0.1 mA cm^−2^	27.8	—	66	Excellent	[92]

*Note:* ORR represents overpotential at a given current density or half‐wave potential (*E*½).

In Table 2, MWCNT/V_2_CT_*x*_ 1.0 M KOH exhibits the lowest HER overpotential with *η*_10_ = 27 mV. Excellent multifunctional performance is demonstrated by CoP@C/Ti_3_C_2_T_x_, which gives trifunctional activity with a HER overpotential of 220 mV in 0.5 M H_2_SO_4_ and an OER overpotential of 235 mV and an ORR half‐wave potential (*E*½) of 740 mV in 1.0 M KOH. Tafel slopes 54–206 mV dec^−1^ and stability exceeding 500 cycles indicate effective interfacial synergy in these MXene‐based hybrid systems. However, MXene surface oxidation and restacking continue to be problems during extended operation, resulting in catalytic deactivation and eventually reducing durability in electrocatalytic applications [44, 93].

## 4 MXenes for Energy Devices and Batteries

Batteries and supercapacitors are among the most popular energy storage devices due to their excellent electrochemical properties. Batteries typically have a high energy density, allowing for long‐term energy supply, but they generally produce less power and are constrained by charge‐storage methods, which can limit their use in microscale or wearable applications. In contrast, supercapacitors provide great power densities and long‐term cyclability, making them ideal for pulse power applications. Despite these advantages, both technologies have intrinsic limitations, underscoring the need for environmentally friendly and cost‐effective materials to improve performance [94]. MXenes have been actively studying energy storage applications since their discovery due to their unusual, layered structures, atomic thickness, large surface area, and superior electrical conductivity. These features enable fast electron transport, short ion diffusion routes, and variable surface chemistry via functional terminations, making MXenes extremely promising for increasing the performance of batteries and supercapacitors. Recent research has investigated MXene‐based composites and hybrid designs, revealing improved electrochemical performance when used as electrodes, electrolytes, and interfaces in energy storage devices, particularly flexible and portable systems [95]. Given their promising structural and electronic properties, MXenes are being investigated as electrode materials for lithium‐ion batteries. For instance, a Ti_3_C_2_T_*x*_ MXene composite doped with sulfur showed a high reversible specific capacity of 393.8 mAh g^−1^ at 100 mA g^−1^ after 100 cycles. This reflects improved lithium storage due to greater interlayer spacing that allows for ion insertion. In comparison to PVDF, utilizing carboxymethyl cellulose (CMC) as a binder improves performance by lowering charge transfer resistance, increasing ion diffusion, and strengthening electrode adhesion. These findings show that MXene‐based composites have high capacity, outstanding cycling stability, and improved rate performance, indicating their promise for future LIB applications [96]. Aside from LIBs, MXene designs have been used in hybrid battery systems like lithium–sulfur batteries (LSBs). MXene‐based designs can decrease polysulfide shuttle and improve cycle stability by providing plentiful active sites and better conductivity pathways. This reduces capacity fading during long‐term cycling. For example, 3D nanostructured Ti_3_C_2_@N‐CNT composites demonstrated an initial discharge capacity of 1214 mAh g^−1^ at 0.2 C, an ultralow decay rate of 0.015% per cycle over 1000 cycles at 1 C, and an aerial capacity of 4.3 mAh cm^−2^ at 4.8 mg cm^−2^ sulfur loading [97]. In addition to lithium‐based systems, MXenes have shown promise as electrode designs in other rechargeable batteries, especially those with multivalent aqueous chemistries like zinc‐ion and aluminum‐ion batteries. High electrical conductivity, tunable interlayer spacing, and rich surface terminations are advantages of MXene‐based cathodes and hybrid composites in aqueous ZIBs. Together, these features enable Zn^2+^ intercalation, buffer the high charge density of multivalent ions, and improve cycling stability over thousands of cycles [98]. MXenes are widely used in batteries, but they also show great promise in supercapacitors and flexible energy devices. By utilizing their metallic conductivity and superior ion accessibility, they can obtain volumetric capacitances of more than 1500 F/cm^3^ [99]. For example, MXene electrodes on a 3D needled and carbonized recycled fiber structure produced ultralight supercapacitors with an energy density of 80.2 μWh/cm^2^ that are perfect for wearables, with a maximum areal capacitance of 1748.5 mF/cm^2^ and >94% retention after 15,000 charge/discharge cycles. By improving mechanical stability, these hybrid designs open the door to sustainable commercial uses [100]. These advancements establish MXenes as frontrunners for next‐generation wearable energy devices, albeit scalable synthesis is still required for commercialization.

## 5 MXenes for Sensors

Sensing technologies are critical in tracking environmental pollutants, detecting harmful gases, diagnosing diseases, and monitoring human health. As a result, there is an urgent demand for robust and efficient sensing systems that can detect specific chemical or biological analytes such as gases, toxic ions, organic compounds, and biomolecules while maintaining high stability, sensitivity, and selectivity for their intended applications [101]. MXenes’ high aspect ratio, rich surface chemistry, and electroconductivity make them appealing for use in sensor manufacturing. High sensitivity, a low detection limit, low fabrication costs, low hysteresis, a fast response with ideally linear and quick recovery for repeated usage are all characteristics of the perfect sensor. High durability over thousands of deformation cycles and a linear response throughout a broad pressure range are essential for pressure and strain sensors. These characteristics have been demonstrated by sensors composed of MXenes, MXene/polymer nanocomposites, and mixed‐dimensional 2D MXene‐based heterostructures [102].

### 5.1 MXenes for Electrochemical Sensors

#### 5.1.1 *Heavy* Metal *Ions*

Heavy metal ions (HMIs) like Pb^2+^, Cd^2+^, Hg^2+^, and Cr^2+^ can linger in the environment for extended periods and pose serious threats to ecosystems and human health. Although conventional techniques such as ICP‐MS, ICP‐AES, and AAS provide high analytical sensitivity, their dependence on costly instrumentation and laboratory operations limits their use for routine environmental monitoring [103, 104]. In this regard, MXene‐based materials have demonstrated outstanding performance as electrochemical sensing platforms owing to their high conductivity, tunable surface chemistry, and large active surface area [105]. Table 3 summarizes representative examples of MXene‐based electrochemical sensors for heavy metal ions reported in recent years, highlighting their compositions, sensor designs, analytical performance, and the remarkable detection capabilities achieved using MXenes.

**TABLE 3:** Comparison of the characteristics of the produced MXene and the synthesis methods for sensing heavy metals.

Synthesis methods	MAX phases and resulting MXenes	Application	Typical SEM and optical morphologies	Structural and surface features	LOD	Ref.
Classical Ti_3_C_2_T_*x*_ synthesis (acid etching + delamination) followed by in situ deposition of Bi nanoparticles.	Ti_3_AlC_2_ → Ti_3_C_2_T_*x*_ → to form BiNPs@Ti_3_C_2_T_*x*_	Electrochemical sensor for Pb^2+^ and Cd^2+^ in water	Accordion‐like multilayer Ti_3_C_2_T_*x*_ sheets decorated with uniformly distributed spherical BiNPs	Surface terminations —O/—OH/—F, micrometer‐scale 2D layered Ti_3_C_2_T_*x*_ flakes with homogenous, continuous morphology, Bi nanoparticles dispersed on the MXene surface.	Pb^2+^/Cd^2+^ simultaneous detection; LODs ≈ 10–12 nM; some Cu^2+^ interference	[103]
Ti_3_C_2_@N‐doped carbon heterostructure obtained by coating Ti_3_C_2_T_*x*_ with N‐doped carbon	Ti_3_AlC_2_ → Ti_3_C_2_T_*x*_ → Ti_3_C_2_@N‐C	Used as an electrochemical sensor for simultaneous Cd^2+^ and Pb^2+^ detection in seawater and tap water	Layered structure where Ti_3_C_2_ flakes are conformally wrapped by thin N‐doped carbon; rough, porous surface	Mixed —O/—OH/—F terminations on Ti_3_C_2_ plus N sites in carbon shell; enhanced charge transfer and selective adsorption; nano/microflakes with preserved crystallinity	Very low LODs (Cd^2+^ 2.55 nM, Pb^2+^ 1.10 nM); excellent resolution and anti‐interference capability.	[104]
Alkali‐intercalated Ti_3_C_2_ (“alk‐Ti_3_C_2_”) prepared via acid etching followed by alkaline intercalation	Ti_3_AlC_2_ → multilayer Ti_3_C_2_T_*x*_ → alk‐Ti_3_C_2_	Used directly as modified electrode for simultaneous Cd^2+^, Pb^2+^, Cu^2+^, and Hg^2+^ monitoring by SWASV	Open accordion‐like Ti_3_C_2_ structure with increased interlayer spacing (c‐lattice expanded from 18.64 to 26.19 Å).	Enhanced —O/—OH terminations due to partial substitution of —F groups, resulting in a more hydrophilic surface useful for ion intercalation.	High sensitivity and low LODs (0.032–0.130 µM) for four heavy metals simultaneously	[106]
LiF/HCl etching + sonication + electrochemical reduction of GO	Ti_3_AlC_2_ → Ti_3_C_2_T_*x*_ → Ti_3_C_2_T_*x*_‐rGO	Efficient electrochemical detection of cadmium and copper ions in water	rGO sheets uniformly covering multilayer Ti_3_C_2_T_*x*_ flakes	Ti—C, Ti—O bonds; reduced oxygen groups; homogeneous Ti/C/O/Al distribution	Cd^2+^ = 0.31 nM Cu^2+^ = 0.18 nM	[107]
HF etching + delamination	Nb_4_AlC_3_ → multilayered Nb_4_C_3_T_*x*_ → probe sonication → delaminated Nb_4_C_3_T_*x*_	Electrochemical detection of Pb^2+^ ions	Wrinkled sheet‐like delaminated flakes	Large interlayer spacing (14.85 Å); high c‐LP (29.70 Å	LOD: Pb^2+^ = 12 nM	[108]
LiF/HCl etching + hydrothermal Sb‐doping	Ti_3_AlC_2_ → Ti_3_C_2_T_*x*_ → 25%Sb@Ti_3_C_2_T_*x*_	Simultaneous electrochemical detection of Pb^2+^, Cd^2+^, and Zn^2+^	Sb nanoparticles (80 nm) on MXene surface/interlayer	Well‐crystallized Sb–MXene composite; increased d‐spacing	Sensitivity Pb^2+^: 3.62 μA·ppm^−1^ Cd^2+^: 2.53 μA·ppm^−1^ Zn^2+^: 0.90 μA·ppm^−1^	[109]
LiF/HCl etching + liquid exfoliated graphene mixing	| Ti_3_AlC_2_ → Ti_3_C_2_T_*x*_ → LEG@Ti_3_C_2_ MXene	Dual‐signal aptasensing for simultaneous Cd^2+^ and Pb^2+^ detection	3D rough layered structure; LEG preventing MXene stacking	Large interlayer spacing; Ti—C/Ti—O/Ti—F bonds; high conductivity	Cd^2+^ = 0.689 pM Pb^2+^ = 1.548 pM	[110]

Table 3 compares seven exemplary MXene‐based electrochemical sensors for heavy metal detection, including early surface modification efforts and more advanced structural designs. Initial techniques, such as BiNPs@Ti_3_C_2_T_*x*_, enabled simultaneous detection of Pb^2+^ and Cd^2+^ with detection limits of 10.8 and 12.4 nM, respectively, mainly due to the enhanced active surface area introduced by Bi nanoparticles [103]. However, interference from Cu^2+^ was detected, likely due to nonspecific adsorption sites. The Ti_3_C_2_@N‐C system showed increased electrochemical performance, with detection limits of 1.10 and 2.55 nM for Pb^2+^ and Cd^2+^, respectively, due to accelerated charge transfer after nitrogen doping [104]. However, the stratified topology limits electrolyte accessibility. More recent research has addressed these restrictions using structural engineering. For example, alkali intercalation [106], has been used to increase the interlayer spacing of MXene, allowing for easier ion diffusion, whilst hybrid frameworks containing rGO or N‐doped carbon suppress MXene restacking and increasing conductivity. Exploring non‐Ti MXenes like Nb_4_C_3_T_*x*_ (limit of detection (LOD) of 12 nM for Pb^2+^) expands the compositional canvas of MXene‐based sensors [108]. Doping techniques, such as 25%Sb@Ti_3_C_2_, enhance sensing capabilities and enable simultaneous detection of Pb^2+^, Cd^2+^, and Zn^2+^ [109]. The LEG@Ti_3_C_2_ aptasensor has the highest sensitivity among known devices, with LOD picomolar level (0.689 pM for Cd^2+^ and 1.548 pM for Pb^2+^) [110]. Overall, the reported studies show that MXene‐based electrochemical sensors with detection limits ranging from μM to pM levels are feasible for heavy metal detection. However, further research is still needed to address issues with long‐term stability and performance in complicated environmental matrices.

#### 5.1.2 Organic Contaminants Sensing

Although MXene‐based electrochemical sensors have proven to be quite effective at identifying heavy metal ions, more recent research has investigated using them to track organic pollutants in food and environmental samples. MXene‐modified electrodes are especially useful for detecting a range of organic pollutants, including toxic gases, harmful volatile organic compounds, and biologically relevant components, because of their high electrical conductivity, hydrophilic surfaces, and tunable surface terminations, which promote rapid electron transfer and provide abundant active sites for molecular adsorption [111]. Wang et al. [112] developed a molecularly imprinted electrochemical sensor with an MXene/polypyrrole (PPy) hybrid for ultrasensitive detection of perfluorooctanesulfonate (PFOS), a persistent organic contaminant that threatens drinking water safety. Controlled pyrolysis produces carbon‐confined, defect‐engineered MXene structures that increase interlayer spacing, inhibit oxidation/restacking, and improve conductivity and electron transfer kinetics, while TiN production increases stability. The sensor has a linear response from 0.02–1000 nM, a limit of detection of 0.5 pM, a relative standard deviation of <5.2%, and recoveries of 90.3–113.3% in tap, river, and simulated wastewater. This technique overcomes MXene's limitations and allows for cost‐effective field environmental monitoring. Tu et al. [113] developed MXene/carbon nanohorns/β‐cyclodextrin‐MOFs for the detection of carbendazim (CBZ) pesticides. In addition to β‐CD‐MOF's host‐guest recognition and porosity for active CBZ adsorption, electrostatic self‐assembly produces MXene/CNHs with better surface area, conductivity, and active sites. With a 3.0 nM–10.0 μM linear range, a 1.0 nM LOD, high selectivity, and effective tomato sample analysis, this synergy demonstrates the adaptability of MXene platforms across a variety of organic contaminants crucial for food safety monitoring. Building on cyclodextrin synergies, Sun et al. utilized MXene–β‐cyclodextrin (MXene–β‐CD) composites for the simultaneous electrochemical sensing of three nitrophenol isomers (*o*‐NP, *m*‐NP, *p*‐NP), persistent nitroaromatic pollutants in environmental waters. Superior selectivity, broad linear ranges (0.4–350 μM for *o*‐NP; 0.5–300 μM for *m*‐NP; 0.5–180 μM for *p*‐NP), ultralow LODs (0.042–0.087 μM), high reproducibility (RSD < 3%), and dependable recoveries (94.6%–103.7%) in lake and tap water were made possible by the host–guest recognition of β‐CD. These findings highlight MXene–CD platforms for multiplexed organic contamination detection that has structural similarities [114]. MXene/NH_2_‐MWCNTs composite, manufactured using electrostatic self‐assembly, efficiently monitors endocrine disruptors such as bisphenols (BPs), which are dangerous compounds commonly found in the environment. The combination of MXene's numerous active sites and NH_2_‐MWCNTs’ better conductivity considerably improved BP sensing compared to individual components. Modified glassy carbon electrodes had wide linear ranges (BPA: 0.1–300 μM; BPB: 0.25–200 μM; BPF: 0.25–150 μM) and low LOD (BPA: 17.53 nM; BPB: 18.43 nM; BPF: 19.32 nM), indicating strong selectivity, repeatability, long‐term stability, and successful real‐world water sample analysis [115]. Furthermore, Sharma et al. improved MXene–rGO nanocomposites as an electro‐immunosensor, which allowed for the ultrasensitive detection of endosulfan, an organochlorine persistent organic pollutant, with LOD of 0.497 ppt (0.1 ppt^–1^ ppm linear range). The DPV‐based platform showed minimal cross‐reactivity, 4‐cycle reusability, and 21‐day stability when MXene's optoelectrical qualities were combined with rGO and in‐house produced anti‐endosulfan antibodies (Ed‐Ab). This demonstrated MXene's ultimate potential for trace‐level environmental pesticide monitoring [116].

### 5.2 Gas Sensors

**FIGURE 10:**
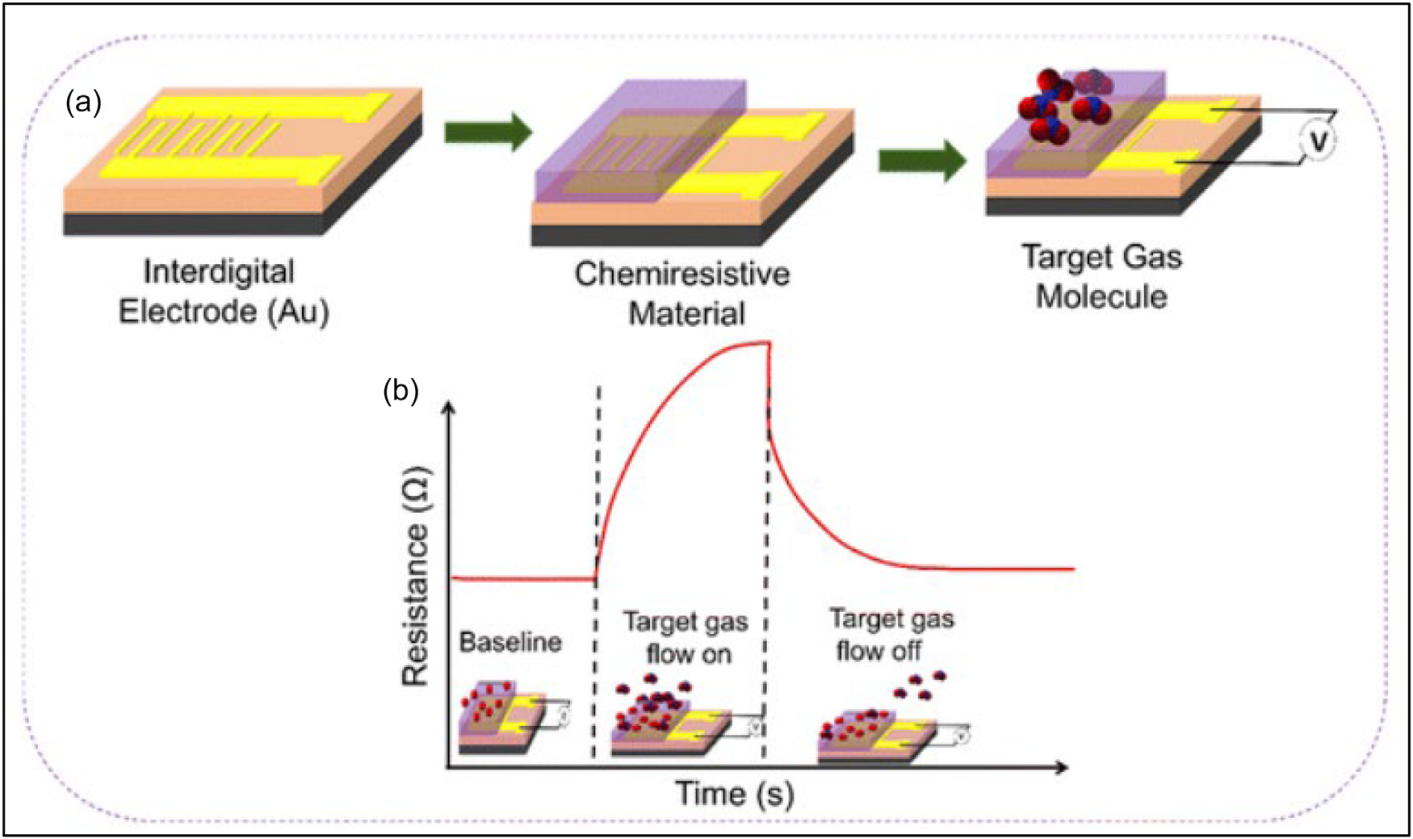
A schematic depiction of the chemiresistive gas‐sensing process, illustrating (a) sensor fabrication procedures and (b) gas molecule adsorption, which increases resistance and then returns to the baseline following desorption. Reproduced from [118] under a Creative Commons Attribution 3.0 Unported License (CC BY 3.0).

Gas sensors are critical components in modern technology because they detect the presence and quantity of gases and turn the data into electrical signals that can be easily monitored and processed. They serve an important role in preserving human health and the environment by allowing for early detection of dangerous or flammable gases in industrial settings, monitoring air pollutants and greenhouse gases in urban areas, and assisting medical equipment that tracks crucial respiratory gases in patients [117]. Gas sensors are one of the most thoroughly researched and widely utilized sensing devices. Their main functioning principle is based on how the electrical characteristics of the sensing material change when it interacts with gas molecules. When gas molecules are adsorbed onto the surface, they change the resistance or current of the material, allowing for exact detection of the desired gas. These sensors are regarded for their ease of manufacture, low cost, ease of use, and potential for downsizing. They can be configured to detect a wide variety of gases, including hazardous and volatile substances. 2D materials have a high surface area, outstanding conductivity, and tremendous sensitivity to surface interactions, making them ideal for building high‐performance and adaptable gas sensors. Gas sensors have been classified into several types based on their working principles, including surface acoustic wave (SAW) sensors, conductometric sensors, and Schottky diode‐based devices. Each category provides various advantages in terms of sensitivity, response time, and operating conditions [101]. Chemi‐resistive gas sensors have received the most attention due to their simple design, low cost, and dependable performance. As shown in Figure 10 the adsorption of gas molecules onto the detecting surface changes the material's electrical resistance, allowing for sensitive detection even at low concentrations. The chemiresistive platform is especially compatible with 2D materials like MXenes, whose high electrical conductivity, large surface area, and tunable surface terminations provide excellent gas‐molecule interactions and allow room‐temperature sensing with enhanced selectivity and stability [118].

Although 2D MXene nanosheets have great electrical conductivity and a high signal‐to‐noise ratio, the tendency to restack after drying causes significant aggregation and a loss of active surface area, limiting their gas‐sensing capabilities. To overcome this issue, the researchers created a flexible room‐temperature NO_2_ sensor using 3D crumpled MXene spheres generated using ultrasonic spray pyrolysis. By incorporating ZnO into the 3D MXene framework, the composite electrode achieved a larger surface area, plentiful active sites, and efficient MXene/ZnO p–n heterojunctions. This resulted in enhanced NO_2_ selectivity, response (27.27%–41.93%), and recovery (∼100%) [22]. The study [20] shows that alkalized Ti_3_C_2_T_*x*_ MXene improves NH_3_ gas sensing performance at ambient temperature. Alkalized Ti_3_C_2_T_*x*_ is a promising MXene platform for NH_3_ gas sensors due to increased surface —O terminations and Na intercalation, resulting in higher response to 100 ppm NH_3_ and good repeatability. Majhi et al. [119] developed a chemi‐resistive sensor using an urchin‐like V_2_C/V_2_O_5_ MXene‐derived hybrid to detect acetone at ambient temperature. The composite sensor surpassed pristine V_2_CT*x* MXene, attaining ppb‐level detection with great selectivity against interfering gases, fast response and recovery, good repeatability, and long‐term stability. The benefits are due to the synergistic interaction of V_2_C MXene and V_2_O_5_, increased surface area, and effective charge transport at the hybrid interface. This study shows how MXene‐derived hybrid structures might help enhance chemi‐resistive gas‐sensing platforms for volatile organic chemicals in environmental and health‐related applications.

## 6 MXene‐Based Solid‐Phase Extractants for Sample Preconcentration

MXenes have been investigated as solid sorbents for sample preparation and heavy metal preconcentration. In one work [120], LiF/HCl etching of Ti_3_AlC_2_ was used to obtain Ti_3_C_2_T_*x*_, which was then bi‐functionalized with amine and thiol groups (NH_2_/SH‐Ti_3_C_2_T_*x*_) by silane condensation in anhydrous toluene. This resulted in an MXene surface that was heavily covered with —NH_2_ and —SH sites. With extraction efficiencies above 97%, limits of detection in the ng mL^−1^ range, and good precision (RSD < 3%), the resultant NH_2_/SH‐Ti_3_C_2_T_*x*_ was used as the solid phase in an ultrasonic‐assisted dispersive microsolid phase extraction (d µ–SPE) procedure for Cd^2+^ and Pb^2+^ in food and soil samples, indicating the potential of functionalized MXenes as effective preconcentration materials for trace heavy metal analysis by FAAS. In a different study [121], unmodified Ti_3_C_2_T_*x*_ MXene was directly used as an adsorbent in solid‐phase extraction procedures for aqueous samples. The high surface area and native surface terminations (–O/–OH/–F) allowed for strong binding of Pb^2+^ and Cd^2+^, as well as low detection limits and good selectivity toward these ions. Overall, these studies demonstrate that both pristine and functionalized Ti_3_C_2_T_*x*_ can be used as flexible solid‐phase materials for preconcentrating hazardous heavy metals before instrumental examination. In addition to heavy metal enrichment, MXene‐based sorbents have also been used to preconcentrate organic micropollutants in complicated matrices. Ti_3_C_2_T_*x*_ MXene nanoparticles were investigated as dispersive solid‐phase extraction adsorbents for multiresidue pesticide monitoring in fruit juice samples prior to HPLC‐MS/MS determination, achieving sub‐μg L^−1^ limits of detection and acceptable recoveries with good precision [122]. Recent reports and reviews on MXene‐based extractants highlight their applicability in various analytical extraction formats (such as dispersive, magnetic, and microsolid‐phase extraction) for environmental and food samples. This highlights MXenes’ versatility as a new generation of high‐performance sorbents for sample preconcentration in analytical chemistry.

## 7 MXenes for Environmental Remediation

### 7.1 Adsorptive Removal

**FIGURE 11:**
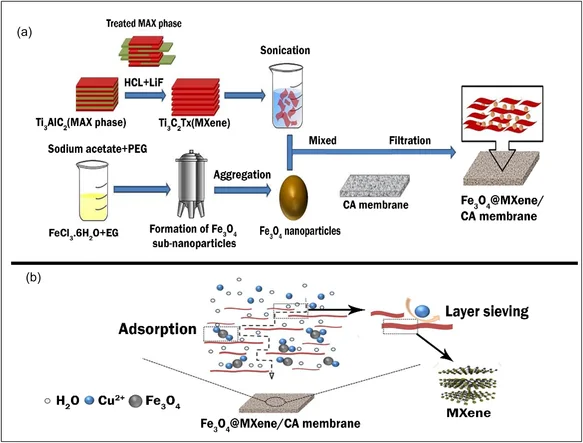
Schematic diagram of (a) the construction of Fe_3_O_4_@MXene/CA nanocomposite NF membrane and (b) separation mechanism. Reproduced with permission [123] copyright 2021, John Wiley & Sons (License No. 6264490071742).

MXenes have adsorption properties that are equivalent to, and in many cases exceed, those of other nanomaterial‐based adsorbents. This superior behavior is due to their large specific surface area, negatively charged surfaces, and abundant surface functional groups (—O, —OH, —F), which together provide numerous active sites for surface complexation, ion exchange, and electrostatic interactions with a wide range of inorganic and organic pollutants. Furthermore, these functional groups can aid in the reduction of some metal ions, increasing the adsorption effectiveness of MXenes against heavy metal pollutants [33]. Representative application of MXenes in adsorption‐assisted separation is demonstrated by Fe_3_O_4_@MXene nanocomposite nanofiltration membranes for heavy metal removal from industrial wastewater, as illustrated in Figure 11. This method expands MXene nanochannels and improves mass transfer by uniformly self‐assembling Fe_3_O_4_ nanoparticles within the 2D MXene lamellar structure on a cellulose acetate platform. Because size sieving and adsorption work in concert, the composite membrane outperforms virgin MXene membranes in terms of water flux and heavy metal removal. For Cu^2+^, 64.1% for Cd^2+^, and 70.2% for Cr^2+^, removal efficiencies of roughly 63.2% are attained. MXene nanosheets create 2D nanochannels for size sieving and surface functional groups for heavy metal adsorption. The use of Fe_3_O_4_ nanoparticles improves adsorption and stabilizes MXene layers. The membrane exhibits good recyclability and performance recovery, demonstrating the usefulness of MXene‐based adsorptive membranes for wastewater treatment [123].

Previous studies on surface functionalization and hybridization have demonstrated that the key to increasing the adsorption capacity is to combine MXene with other materials based on their ability to enhance each other synergistically. In this case, MXene can be thought of as an electron acceptor and conductive substrate to achieve the quick charge transfer that results in the redistribution of electrons [50]. Recent research found that MXenes may be employed efficiently as adsorbents for the removal of heavy metal ions and organic dyes from wastewater. TiVCT_*x*_ MXene was produced using in situ etching of a TiVAlC precursor and tested for the adsorption of Cr(VI) and methylene blue. TiVCT_*x*_ nanosheets demonstrated strong adsorption capabilities at different pH levels. Under acidic conditions, Cr(VI) adsorption followed the Langmuir isotherm and pseudo‐second‐order kinetics, indicating monolayer chemisorption dominated by reduction; under alkaline conditions, MB adsorption followed the Freundlich isotherm and pseudo‐second‐order kinetics, driven by electrostatic interactions and hydrogen bonding. The nanosheets also demonstrated quick adsorption kinetics and great resistance to ionic interference, indicating their practical utility in wastewater treatment applications [124]. MXenes have been used as adsorbents to remove antibiotics such as ciprofloxacin and tetracycline from contaminated effluents. In this study [125], SI‐Ti_3_C_2_T_*x*_ MXene is described as an effective adsorbent for ciprofloxacin, with a maximum adsorption capacity of about 223.8 mg g^−1^ driven by chemical interactions between —O, —OH, and —F terminations and the antibiotic. The scientists used NaCl to combine adsorption with electrochemical regeneration in a divided cell. They achieved complete regeneration over ten cycles at 787.5 C g^−1^, 7.5 min, 10 mAcm^−2^, and 2.5 w/v% NaCl. Structural investigations demonstrate that regeneration preserves the MXene framework while increasing defect density and oxygen/nitrogen‐containing groups, thereby doubling adsorption capacity [125]. By combining pure MXenes with extremely porous frameworks like ZIF‐8, some of their inherent drawbacks, including their relatively low accessible surface area, undeveloped porosity, and propensity for nanosheet restacking, can be partially addressed. As shown for MXene@ZIF‐8 hybrids, ZIF‐8 can be intercalated between Ti_3_C_2_T_*x*_ layers and surface terminations (—O/—OH) can be tailored to create multilayer, highly porous architectures with significantly stronger noncovalent interactions toward organic dyes and antibiotics. This unlocks the potential of MXene‐based adsorbents for effective water treatment [126].

### 7.2 Decontamination and Detoxification

MXene‐based products have shown promises for environmental decontamination due to their antibacterial capabilities. The prevalence of harmful microorganisms in water systems poses a substantial barrier to water purification technology. Delaminated Ti_3_C_2_T_*x*_ MXene has shown outstanding antibacterial action against Gram‐negative Escherichia coli and Gram‐positive B*acillus* subtilis, achieving around 98% bacterial inactivation at a concentration of 200 μg mL^−1^ after 4 h. Rasool et al. discovered that the sharp edges of MXene nanosheets can physically break bacterial membranes, resulting in the release of cytoplasmic components and final cell death. These antibacterial capabilities emphasize MXenes’ potential for reducing microbial contamination and biofouling in water treatment systems [127]. Recent studies suggest that additional mechanisms, including ROS production and electrostatic interactions, may contribute to the broad‐spectrum antimicrobial activity of MXenes against various microorganisms [128]. Overall, these characteristics make MXenes promising materials for improving the efficiency and stability of water purification technologies. Beyond microbiological elimination, MXene‐based products can help with environmental detoxification by lowering the toxicity of harmful chemicals. Several studies have shown that MXene composites can immobilize heavy metal ions and facilitate the transition of dangerous organic chemicals into less damaging forms, thereby reducing their environmental impact and improving water quality [129]. For example, recent research has shown that MXene‐based composites can successfully remove hazardous heavy metal ions from contaminated water. A Fe_3_O_4_@MXene nanocomposite effectively removed Cu^2+^ ions from wastewater, with removal efficiencies of over 83% [130]. MXene‐based materials have also shown promise for environmental detoxification by lowering the toxicity of harmful contaminants. Monolayer Ti_3_C_2_T_*x*_ nanosheets can successfully detoxify hexavalent chromium (Cr (VI)) in water, converting it to the less dangerous Cr (III) form. The reduction happens predominantly through OH^–^ terminals on the MXene surface. The resultant Cr (III) is stabilized through complexation with oxygen‐containing groups. This combination of reduction and surface anchoring greatly lowers the environmental and biological dangers of Cr (VI), emphasizing the promise of MXene‐based materials for rapid and efficient water detoxification [131]. This highlights the promise of MXene‐based materials for lowering heavy‐metal toxicity in aquatic environments.

## 8 Challenges and Future Perspectives

Despite significant progress in MXene synthesis and applications, long‐term stability is still a critical problem, especially in aquatic conditions where many energy storage and solution‐processable devices are used. MXenes are very vulnerable to oxidation and hydrolysis caused by interactions with water molecules and dissolved oxygen, which result in the development of transition metal oxides and a loss of electrical conductivity and functional characteristics. In addition to nanosheet restacking, which lowers exposure of the active site during catalysis [93]. Experimental and theoretical studies demonstrate that water itself plays a dominant role in starting deterioration, even in the absence of oxygen, by attacking the MXene surface and driving hydrolysis processes that undermine structural integrity [132]. Fundamental experiments have confirmed that storage conditions such as temperature, dissolved oxygen content, and solvent environment have a significant impact on oxidation and degradation. Lowering the storage temperature and removing oxygen can significantly prevent the oxidation process, while substituting aqueous medium with organic solvents like dimethylformamide (DMF) or propylene carbonate (PC) lowers interaction with water and increases stability. However, these solutions frequently require additional processing complexity or constraints in dispersibility, and they are not universally applicable to all MXene systems [133]. Recent work on long‐term storage approaches, such as the use of antioxidants, for example, sodium L‐ascorbate in MXene dispersions, demonstrates that oxidative degradation can be delayed over long periods with minimal loss of electrochemical performance, indicating the potential for more cost‐effective and scalable stabilization strategies [134]. Other methods, such as defect passivation and edge capping with polyanionic salts, have been demonstrated to improve oxidation resistance by lowering reactive edge sites that act as nucleation centers for oxide formation [135]. Edge‐passivated Ti_3_C_2_T_*x*_ and V_2_CT_*x*_ MXenes with polyanionic salts improve oxidation stability by blocking water molecules and dissolved oxygen that induce oxidation [136]. Other difficulties include the limited study of prospective MXene variations other than Ti_3_C_2_T_*x*_ and Mo_2_CT_*x*_, such as double‐transition metal and V‐based MXenes, and the scalability constraints of existing fluorine‐based synthesis methods that impede industrial manufacturing [44].

Despite the remarkable progress in the development and application of 2D MXenes (2DMxn), several scientific and technological challenges remain that must be addressed to fully realize their potential in advanced functional devices. Future research should therefore focus on scalable and environmentally safe synthesis routes, particularly fluoride‐free and green approaches that can enable the large‐scale production of high‐quality MXenes while minimizing environmental impact. Another important direction is the precise control of surface chemistry and interlayer engineering, since surface terminations strongly influence the electronic structure, catalytic activity, and adsorption behavior of MXenes. In this context, advanced surface modification strategies, such as ALD, heteroatom doping, hybrid nanostructure formation, and interface engineering, are expected to further improve the functionality and durability of MXene‐based systems.

In addition, future efforts should emphasize the design of 3D architectures, porous frameworks, and composite structures that prevent restacking, maintain high electrical conductivity, and preserve active‐site accessibility. Incorporating MXenes into hybrid materials with polymers, carbon‐based materials, or metal oxides may also enhance mechanical stability and electrochemical performance. MXenes are expected to play an increasingly important role in next‐generation batteries, supercapacitors, and electrocatalytic systems, making it essential to better understand the structure–property–performance relationships, particularly the mechanisms governing ion transport, charge transfer, and catalytic reactions at MXene surfaces. Advanced in situ and operating characterization techniques will be crucial for revealing these mechanisms and guiding rational material design.

MXene‐based sensing and environmental technologies also represent a promising area of future research. Their high electrical conductivity and abundant surface functional groups make them highly sensitive platforms for chemical and biological detection, and further work should focus on selective, portable, and real‐time sensing devices for environmental monitoring and healthcare diagnostics. From an industrial approach, the development of high‐yield synthesis pathways, batch‐to‐batch reproducibility, process automation, efficient waste management, and steady access to precursor materials are all necessary for the scalability of MXene production. Large‐scale implementation still necessitates careful quality control, cost optimization, and compliance with changing environmental and safety regulations, but scalable routes like LiF‐HCl‐based etching are particularly appealing because they are comparatively accessible and less dangerous than traditional HF‐based methods. To facilitate the shift from laboratory synthesis to commercially viable manufacture, a practical industrial strategy should also take supply‐chain stability, process engineering, and life‐cycle cost analysis into account. Finally, bridging the gap between laboratory‐scale research and practical deployment remains a key challenge, so future efforts should emphasize scalable manufacturing, device integration, and long‐term stability testing under real operating conditions. Collaboration between materials scientists, chemists, engineers, and industry partners will be essential to accelerate the commercialization of MXene‐based technologies.

## 9 Summary and Outlook

**FIGURE 12:**
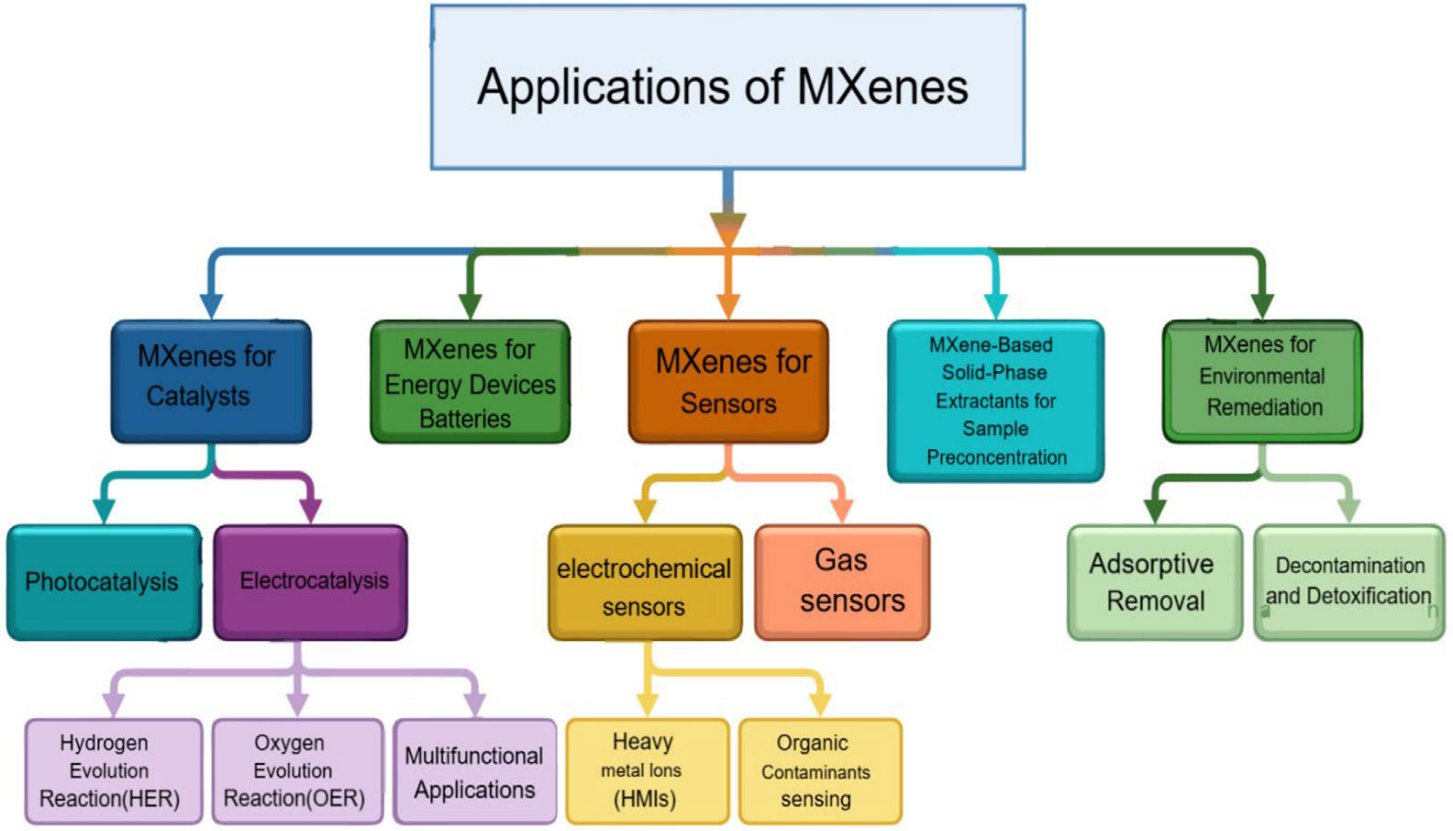
Schematic illustration of the main application of MXenes.

In summary, MXenes have rapidly evolved from HF‐etched Ti_3_C_2_T_*x*_ prototypes into a broad family of 2D transition‐metal carbides, nitrides, and carbonitrides synthesized via diverse top‐down and bottom‐up routes, including fluoride‐based, fluoride‐free, molten‐salt, CVD, template‐assisted, and mechanochemical strategies. Advances in synthesis and postprocessing, including solvothermal decoration, microwave‐assisted modification, ALD, electrochemical engineering, and ball‐milling, have allowed for precise control over phase composition, interlayer spacing, surface terminations, and hierarchical architecture. These factors influence electrochemical, catalytic, and adsorption performance. MXene‐based materials play important roles in photocatalysis, electrocatalysis (HER/OER/ORR), energy storage devices (batteries, supercapacitors, flexible power sources), sensors (electrochemical and gas sensors), solid‐phase extractants, and environmental remediation platforms, as summarized in Figure 12. MXene research will likely shift from proof‐of‐concept studies to scalable, application‐driven design. Priority directions include (i) safer, fluorine‐free, and molten‐salt etching protocols compatible with industrial‐scale production; (ii) exploration of under‐studied compositions such as double‐transition‐metal and V‐based MXenes; (iii) rational surface and defect engineering to decouple high activity from oxidation‐induced degradation; and (iv) construction of multifunctional heterostructures capable of simultaneously delivering catalysis, sensing, and charge storage. Combining MXenes with data‐driven screening, in situ/operando characterization, and device‐level optimization (e.g., MXene‐enabled μPADs, flexible electronics, and IoT‐connected platforms) will accelerate the translation of MXenes from laboratory materials to robust technologies for sustainable energy and environmental applications monitoring.

## 10 Conclusion

2DMxn have rapidly emerged as a highly promising class of materials with exceptional physicochemical properties, including metallic conductivity, large surface area, tunable surface terminations, and strong hydrophilicity. These characteristics make MXenes attractive candidates for catalysis, sensing, environmental remediation, and energy storage and conversion technologies. This review has summarized the major progress achieved in MXene research since 2020, from synthesis strategies to advanced functional applications. Although significant advances have been made in top‐down and bottom‐up synthesis, as well as in postsynthesis modification, several challenges remain regarding stability, scalability, and structural control. Addressing these issues through improved synthesis routes, surface engineering, and stronger material integration will be essential for further development. Overall, MXenes represent a transformative platform for next‐generation functional materials and are expected to play an important role in sustainable energy, environmental monitoring, catalysis, and sensing technologies in the near future.

## Author Contributions

All authors contributed to the literature review, manuscript drafting, editing, critical revision, and approval of the final version.

## Funding

This research work was funded by Umm Al‐Qura University, Saudi Arabia under grant number: 26UQU4290372GSSR04.
